# Abstract Sifter version 8: Focus on the chemical literature

**DOI:** 10.12688/f1000research.160617.2

**Published:** 2026-06-06

**Authors:** Nancy C. Baker, Thomas B. Knudsen, Antony J. Williams

**Affiliations:** 1 ParlezChem, Hillsborough, North Carolina, USA; 2 Department of Intelligent Systems Engineering, Luddy School of Informatics Computing and Engineering, Indiana University, Bloomington, Indiana, USA; 3 Center for Computational Toxicology and Exposure, Office of Research and Development, U.S. Environmental Protection Agency, Research Triangle Park, North Carolina, USA

**Keywords:** Literature mining, knowledge mining, PubMed, DSSTox, drug discovery, toxicology

## Abstract

Effective research depends on building on the knowledge found in the scientific literature. Designed to streamline literature tasks, the EPA’s Abstract Sifter literature tool, now at version 8, has been continually extended and enhanced since its introduction in 2017[1]. Early enhancements to the tool have primarily focused on core tasks common to all researchers. For example, citation retrieval from PubMed has been made faster and the returned citation threshold increased to 10,000. Features that allow deeper examination of the literature have been introduced as well. A functionality called Term-mapping allows for fast, dynamic relevancy ranking of returned citations. MeSH substances, such as proteins, genes, and chemicals, can now be extracted from a retrieved corpus of citations, ranked by frequency and explored through the MeSHMine functionality. Features that facilitate user engagement with publications have also been improved: formatting and colorization ease reviewing of the abstract text and the tagging and noting citations functionality has been streamlined. Version 8 introduced multiple features that break new ground in working with chemical literature. For example, chemical entity extraction from scientific publications has been streamlined through download of PDFs and automated table extraction. Following entity extraction, the chemical names can be used as inputs to retrieve EPA’s chemical identifiers, the DSSTox (Distributed Structure-Searchable Toxicity) chemical IDs (DTXSIDs). Once these identifiers have been retrieved, a wealth of chemical information is available through built-in functions accessing EPA’s Computational Toxicology and Exposure application programming interface (CTX-APIs) [2]. This new functionality allows researchers to build on the EPA’s efforts in chemical data assembly and curation. The Abstract Sifter version 8 is a valuable tool for researchers endeavoring to understand chemicals and their effects on the environment and biological systems.

**Abbreviations**
API: Application Program Interface – calls made to data stores
AS: Abstract Sifter
CAS RN: Chemical Abstract Service registry numbers
CCTE: EPA’s Center for Computational Toxicology and Exposure
ChEBI: EMBL Chemical identifier
CTX-API
: CCTE Comptox APIs
DSSTox: Distributed Structure-Searchable Toxicity database – EPA’s chemical database
DTXSID: DSSTox substance identifier -unique identifier for chemicals in the DSSTox database. Distributed Structure-Searchable Toxicity database identifiers
EMBL EBI: European Molecular Biology Labs, European Biotechnology Institute
EPA: United States Environmental Protection Agency
MeSH: Medical Subject Headings – controlled vocabulary used by NLM
NCBI: United States National Center for Biotechnology Information
NLM: United States National Library of Medicine
PDF: Portable Document Format
RIS: Research Information Systems – a format for citation exchange
SMILES: Simplified Molecular Input Line Entry System
VBA: Visual Basic for Applications – programming language used in Windows applications

## Introduction

Efficient and effective use of biomedical literature helps ensure that researchers are finding and building on previously published research. For nearly 10 years, the EPA’s Center for Computational Toxicology and Exposure (CCTE) has been developing the Abstract Sifter (AS) tool to support its research and has made the tool publicly available. ^1^ The novel design of the AS combines API calls for delivering public data with the versatility of a Windows Excel macro-enabled workbook.

The technologies that AS relies on – Microsoft Excel, its associated Visual Basic for Applications (VBA) programming language, and available public APIs – have themselves improved over the years. For example, the richness of Excel’s capabilities has expanded, an increasing number of APIs have been released to the public, and APIs deliver more data over time. This combination gives the researcher, reader, and practitioner a wealth of easily navigable information to build on.

This publication reviews the features of AS with a focus on the enhancements added in version 8, the latest version. The content is organized according to the literature tasks that the tool facilitates. These tasks include querying PubMed and retrieving citations, building a corpus of articles, and the important task of ranking them by relevancy using the sifting and term-mapping functionalities. User engagement with documents and how the AS facilitates this task with colorization of text, tagging, and note-taking is also covered. We present a new functionality -data extraction- and demonstrate how the user can extract tables and text and convert them into data for analysis or for feeding into new studies using AS. The section regarding data extraction highlights the new emphasis on extracting chemical entities. The collection of chemical entities is followed by discussion of chemical sets: how to build, extract, expand, and analyze them.

**Figure 1.**
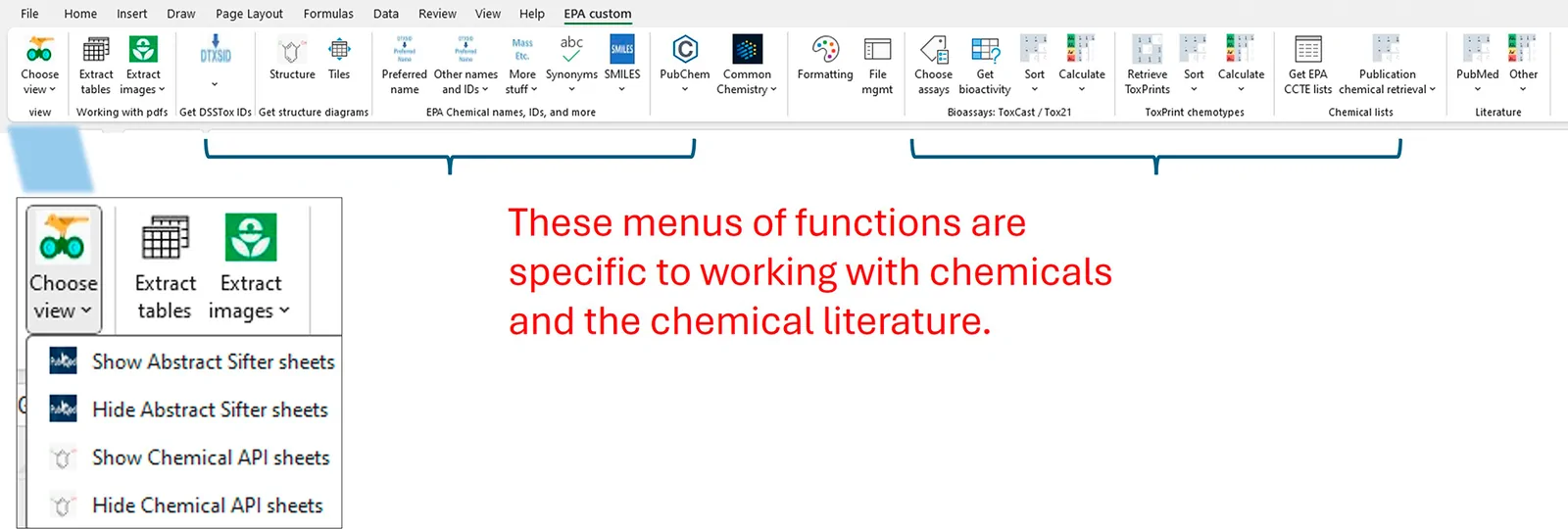
Abstract Sifter version 8 introduces a new EPA Custom ribbon that organizes the new functionality. The Choose view section hides or unhides certain sheets to help the user simplify the workplace. To see the sheets discussed here, toggle the sheets hidden/shown or manually unhide a sheet of interest.

With the new diverse functions in AS, keeping the user experience positive has been a priority. Many of the new functions have been packaged into a new custom EPA ribbon. The ribbon has options to hide or unhide sheets to streamline and unclutter the AS workbook ( Figure 1).

This publication is not a how-to guide and for specific instructions the reader is encouraged to refer to the user guide or watch any of the available videos. Abstract Sifter version 8 with the user guide is available at https://www.parlezchem.com/abstract-sifter/abstract-sifter-version-8/. The ReadMe sheet of the Abstract Sifter has links to how-to videos. Searching on YouTube with “abstract sifter” will retrieve video demonstrations from the user community.

## Methods

### Implementation

AS is an Excel macro-enabled workbook. ( https://www.microsoft.com/en-us/microsoft-365/excel) It contains VBA code that retrieves data from public API-enabled data sources (e.g., PubMed and PubChem). VBA routines also control user interaction through menus, forms, formatting, and navigation.

AS contains specialized sheets with specific functionality attached to them. The core sheets introduced in AS version 1 were Main, Abstract, Log, Notes, and Landscape. Version 8 adds additional sheets for the newly introduced functionality: ToxPrint and Bioassay sheets. Additional sheets are created when the user executes particular functions (e.g., Tiles and ChemLists). The new EPA custom ribbon ( Figure 1) helps with navigation of the expanded functionality and additional sheets.

### Operation

AS requires Microsoft Excel running on a Windows platform. The BioAssay and Toxprints functionality require Excel 2007 or newer versions. Some formatting options require Excel 2013 or newer versions. Users whose site requires a signed certificate should contact the first author.

## Use cases

### Querying PubMed, retrieving, and ranking citations

The Main sheet of AS offers the most direct interaction with PubMed content. In this worksheet, a user enters a query that gets sent to the NCBI NLM e-utility APIs to retrieve the results. Each returned citation is inserted into one row of the Main sheet. The record limit for one query’s retrieval is 10,000 records, but larger corpora can be built by running multiple queries and appending the results through selecting the Append option before running another query. Any duplicate records can be deleted using a function called from a button on the Main sheet. There is no limit to the number of queries that can be run. Each query that is run to completion is recorded on the Log sheet along with the number of records returned, date and time run, and the time elapsed to retrieve the records. Double-clicking on a row on the Log sheet will rerun the query.

Inserting natural language titles and abstracts into rows in a spreadsheet treats these elements like data and, as a result, built-in Excel capabilities facilitate sorting and filtering of the results. Even with the built-in Excel filtering, finding relevant records in a large corpus is a challenge. For many literature projects, the goal of the user may be exploratory: reading and learning, exploring nascent ideas and adjusting course following the exploration. AS facilitates, encourages, and helps organize dynamic exploratory research.

**Figure 2.**
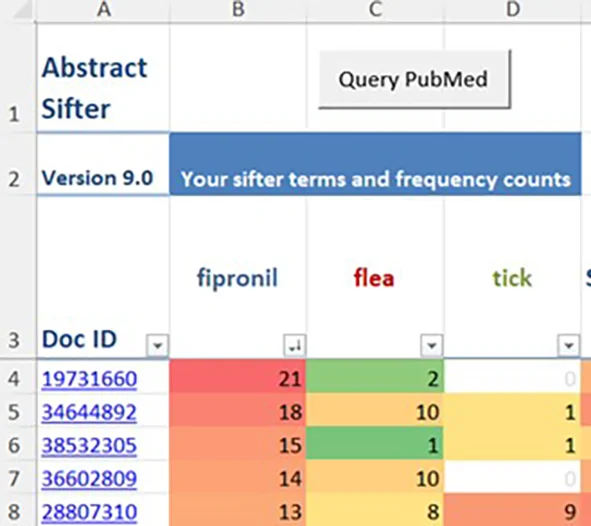
View of the Sifter cells on the Main sheet.

Using previous versions of AS, a researcher’s exploration of a literature corpus was enabled through the “sifting” process. The Main sheet of the AS has three cells where the user can enter a string of characters and the AS, through a built-in formula, will count the occurrences of the string in the titles, abstracts, and keywords of the Main sheet records ( Figure 2). Sorting based on these counts ranks the records by that concept. This action can be repeated as often as desired with the combination of the three sifter cells providing visual insight into a citation’s current relevancy.

A common question from early users of the AS was “Can I sift on more than three terms at a time?”. With the term-mapping functionality, this is possible. Term-mapping allows the user to find, count, and sort on tens or hundreds of terms and then rank the Main sheet corpus by the total number of mapped terms. This functionality starts with the TermMap sheet. Here term lists can be built or downloaded from a collection of starting point terms organized by common chemical literature queries. To describe this feature using text-mining and machine learning terminology, the TermMap sheet can be seen as a feature set and the count of the features will indicate a record’s relevance.

**Figure 3.**
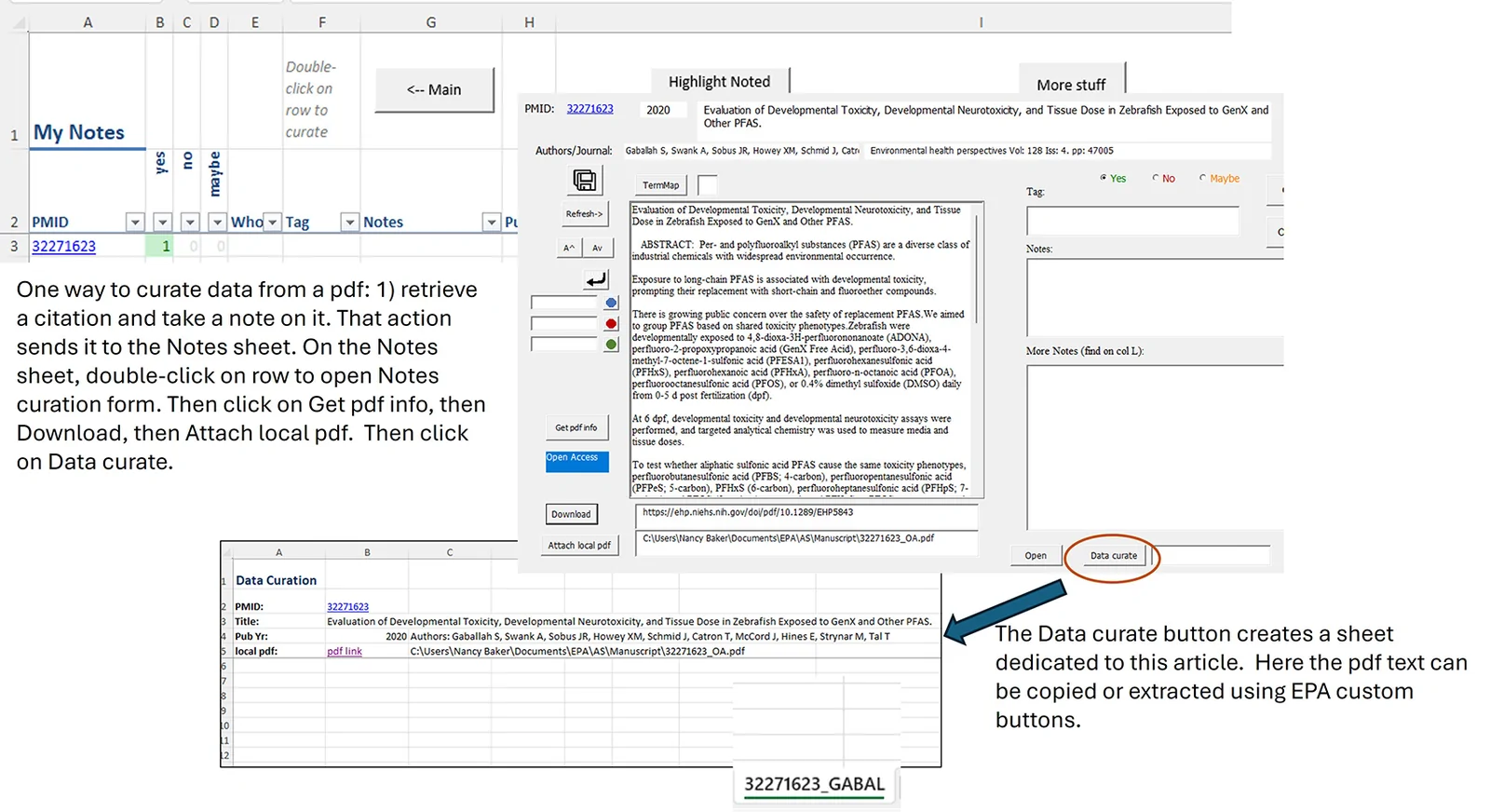
a and b. Examples of ranking citations by term mapping.

In the example shown in Figure 3, the simple query “chlorpyrifos” was run (accessed 09/20/2024) and the resulting 6900 PubMed records inserted on to the Main sheet. On the TermMap sheet, the term list for “environmental fate” was selected, downloaded, and then mapped to the chlorpyrifos articles using the “Map!” Button ( Figure 3a) Following mapping, the Main Sheet articles can be ranked by the total number of included fate concepts and the most relevant articles regarding environmental fate rise to the top. The TermMap sheet makes this easy: double-clicking on the column A value writes this term prefixed with “TX:” to the Main sheet’s B3 sifter cell and sorts. In the second example, a term list related to “analytical methods” used for the purpose of chemical identification and measurement was mapped ( Figure 3b). Double-clicking on “chemid” ranks the entire corpus by the number of listed chemical identification terms found in each citation.

The term-mapping functionality is a powerful way to quickly find relevant articles of interest. With term-mapping, the user is no longer reliant on long complex queries to find a subset of articles. Instead, the user can use simple queries less prone to error and then apply term-mapping to rank the corpus by the desired features. Using AS, there is no need to employ computationally expensive and often opaque machine learning technologies.

### Working with literature citations: Reading, tagging, note-taking, curating notes

Using AS, researchers can read and engage with article content in several ways: skimming titles and abstracts, filtering and screening articles of interest, attaching categorization tags, and adding notes summarizing an aspect of the article that can be referred to later or shared with colleagues. The AS facilitates all these forms of engagement through the Notes functionality. Taking notes, either from the Main sheet or from the Abstract sheet, copies a citation’s information to a row on the Notes sheet. The user can then add tags or text to the record or click on the *Yes*, *No* or *Maybe* categorization button. The Notes sheet therefore provides a place to store selected citations and build a corpus of publications.

**Figure 4.**
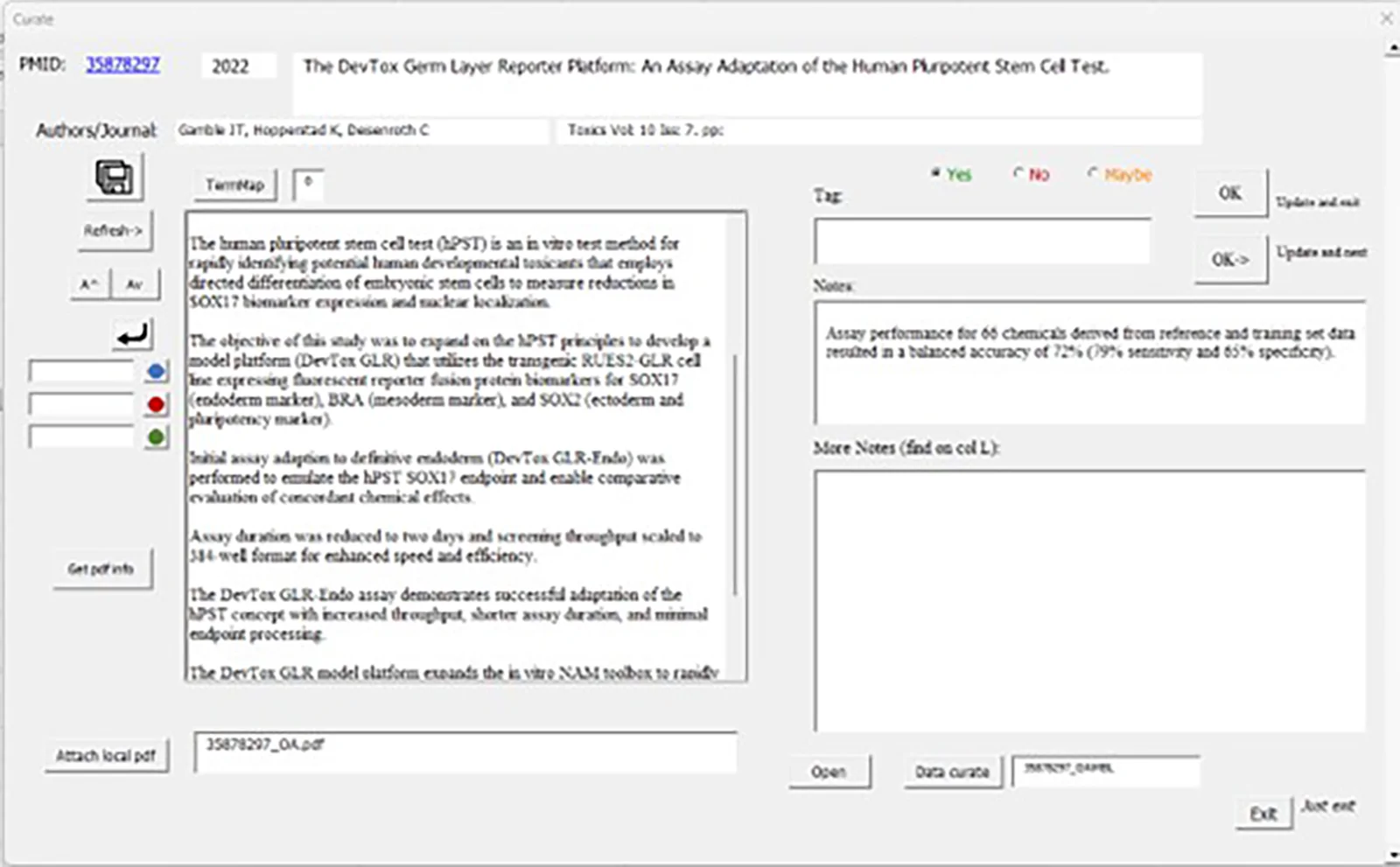
Double-click on a row on the Notes sheet to work with Notes curation form.

Double-clicking on a row in the Notes sheet activates the Notes curation form ( Figure 4). AS version 8 introduces several enhancements to notes curation, including usability features (e.g., larger font, adding line feeds, colorization), drag and drop tagging and noting, and PDF retrieval. The curation form includes a function from Unpaywall ^3^ that checks to see if the PDF is open access and, if so, can be downloaded with a local link added to the Notes row. The Notes curation form is the entry point to curating data from the article itself. Clicking on the *Data Curate* button will create a new sheet specific for that article named with the PubMed ID and part of the first author’s name. Here, on this dedicated sheet, the user can extract, store, and work with data from a publication.

New importing and exporting functions have been added. Functions are available through Main and Note sheet buttons that import from and export citations to various formats including RIS (Research Information Systems), a format that can be used as input into citation management software such as EndNote. Citation information and notes can be exported as a text file which can then be worked into text for a manuscript.

### Extracting chemical entities from the literature

A common task in biomedical research is to find the chemical entities associated with a particular disease or biological process and then conduct further research on those chemicals. Named entity extraction (NER) is the task of finding entities like chemicals in scientific literature, extracting the entity name from the text, and associating that name with an external unique identifier that maps to the entity contained within a public database.

Because of the importance of entity extraction, several scientific groups have been extracting chemical entities from scientific articles and making them available to the community. Foremost among the groups working in this domain is the US National Library of Medicine (NLM), the organization responsible for the development of its MeSH term annotations. ^4, 5^ MeSH terms are a controlled vocabulary in a tree structure that are attached to most PubMed citations. MeSH annotations encompass many types of entities including diseases, anatomical terms, proteins, methods, and chemicals. PubChem, another database under the NLM and the National Center for Biotechnology Information (NCBI), uses various automated and deposition-based methods to identify chemicals in publications, associate them with PubChem identifiers, and make them available. ^6– 8^

The US EPA has recently started an effort to curate chemicals from publications. This project has the goal of extracting chemical entities and, using a combination of expert curation and computational methods, associate the chemical name (or other identifiers) with the DTXSID. ^9^ The DTXSID is the primary identifier associated with the chemical registration database hosting data associated with many of the applications from the Center for Computational Toxicology and Exposure (CCTE), including the CompTox Chemicals Dashboard (referred to henceforth as CCTE Dashboard). ^10^ The publication identifier and the associated DTXSIDs are stored in a database for the purposes of search and retrieval via one or more software applications. The project was originally initiated with the intention of assembling documented analytical methods assembled from various sources, extracting chemicals within the documents, and making the analytical methods searchable by chemicals identifiers and structures. AMOS, the Analytical Method and Open Spectra database, is the web-based application delivering over 6,000 methods indexed by chemical structure. AMOS is intended to be released to the community in the near future.

AS version 8 and AMOS both use the new CCTE publicly accessible CTX-APIs. ^2^ The documentation is provided here: https://api-ccte.epa.gov/docs/index.html.

*MeSH Mining*

AS implements several approaches to entity extraction. The first approach, MeSH Mine, extracts MeSH substance annotations from a corpus of citations on the Main sheet. MeSH terms are a controlled vocabulary of terms maintained by NLM and attached to PubMed citations. The methods used to find and assign MeSH terms have evolved over the years from manual annotation by subject experts to a growing reliance on automated text-mining. ^4, 5^

**Figure 5.**
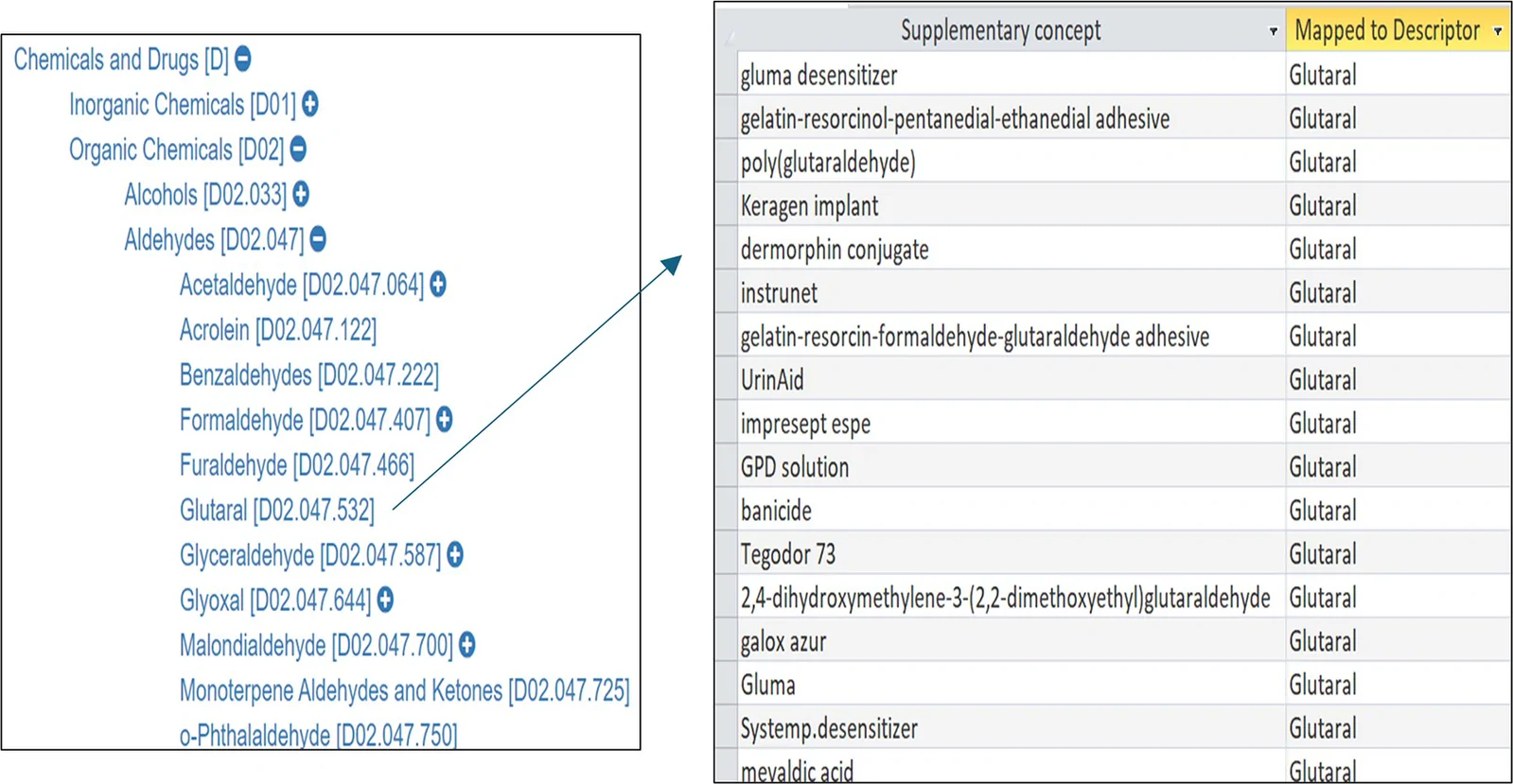
A section of the MeSH tree showing chemicals and chemical families. On the right, an illustration of how chemical entities are mapped to a chemical or family on the MeSH tree.

The MeSH vocabulary encompasses two types of chemical terms. The first type, called Descriptors, includes chemical families and chemical names that are a part of the MeSH tree branch for chemicals; however, most chemicals are entered as the second type of terms – Supplemental concepts – into MeSH and have a mapping back to a chemical or chemical family in the MeSH tree. In the example in Figure 5, a section of the Organic Chemicals branch is shown. On the right is a subset of the chemicals mapped to Glutaral, a chemical in the branch. Collectively, the chemical descriptors and chemical supplementary concepts are called substances.

**Figure 6.**
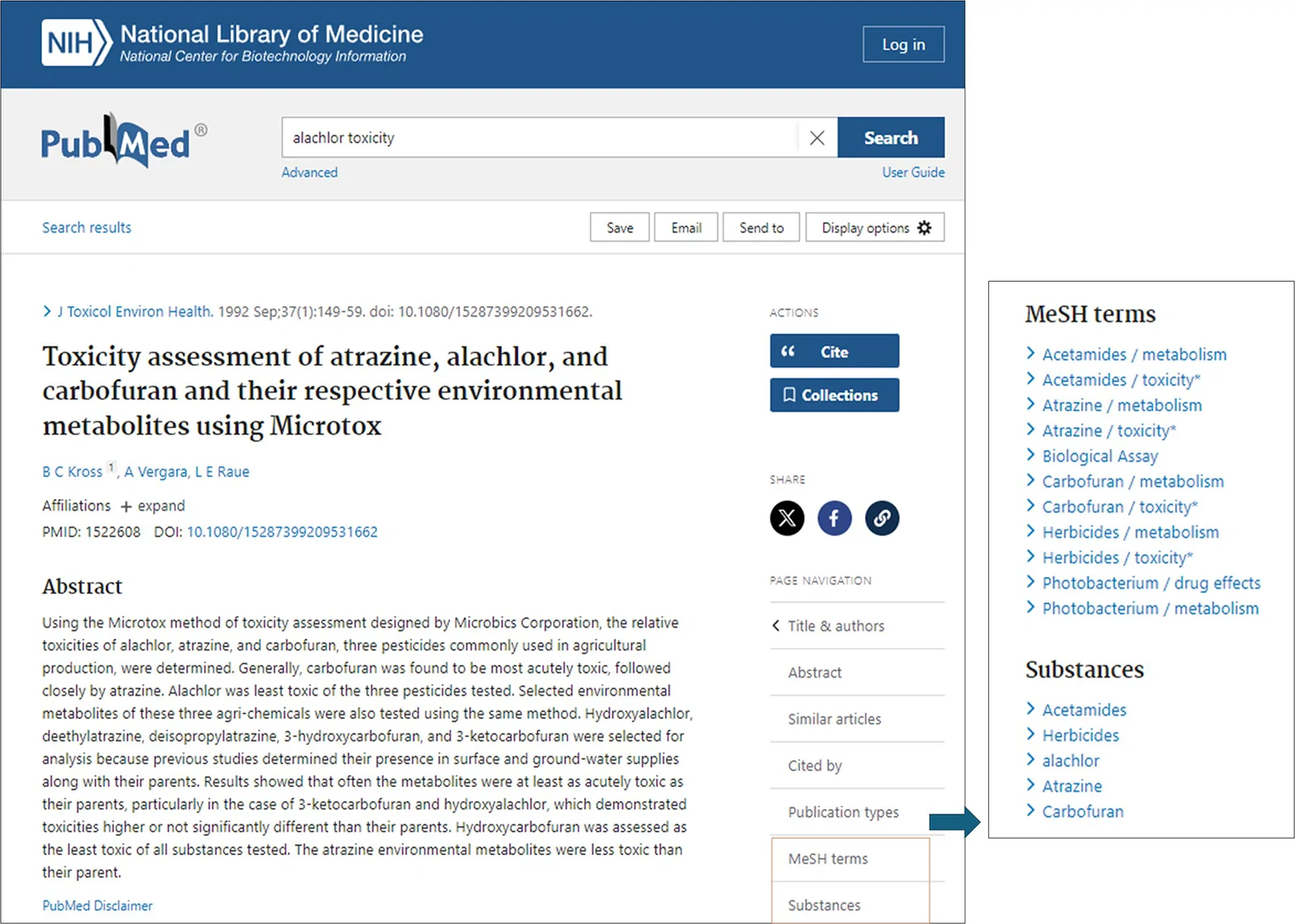
In PubMed, the MeSH term and substance annotations are found by clicking on the link to the right.

In PubMed, the MeSH term annotations can be viewed by clicking on the MeSH Terms link to the right of the page ( Figure 6). For example, the annotations include chemicals, chemical families, study methods (Biological Assay) and species (Photobacterium).

With AS, MeSH substance terms can be extracted from the retrieved and downloaded citations, organized, counted and placed on a new sheet: MeSHMine. To understand the power of this functionality an example query can be executed on the Main sheet query form: *Cornea [majr] AND chemicals.* The query results in over 2600 entries written to the Main sheet. Clicking on the *More things* button, followed by *Extract MeSH substances buttons* prompts AS to search each row on the Main sheet for the MeSH substance terms associated with the citation by NLM, count their occurrences, and write them to the MeSHMine sheet. When this is completed, the MeSHMine sheet holds a wealth of information and helps the researcher navigate through the data. The MeSHMine sheet is organized based on the substance name listed in column A and hyperlinked to the MeSH browser. Flags indicating the class of chemical substance (i.e., organic, inorganic, macromolecule, lipid, protein, etc.) are listed in the columns to the right. The researcher can select a subset of chemicals of interest by sorting or filtering on these columns.

**Figure 7.**
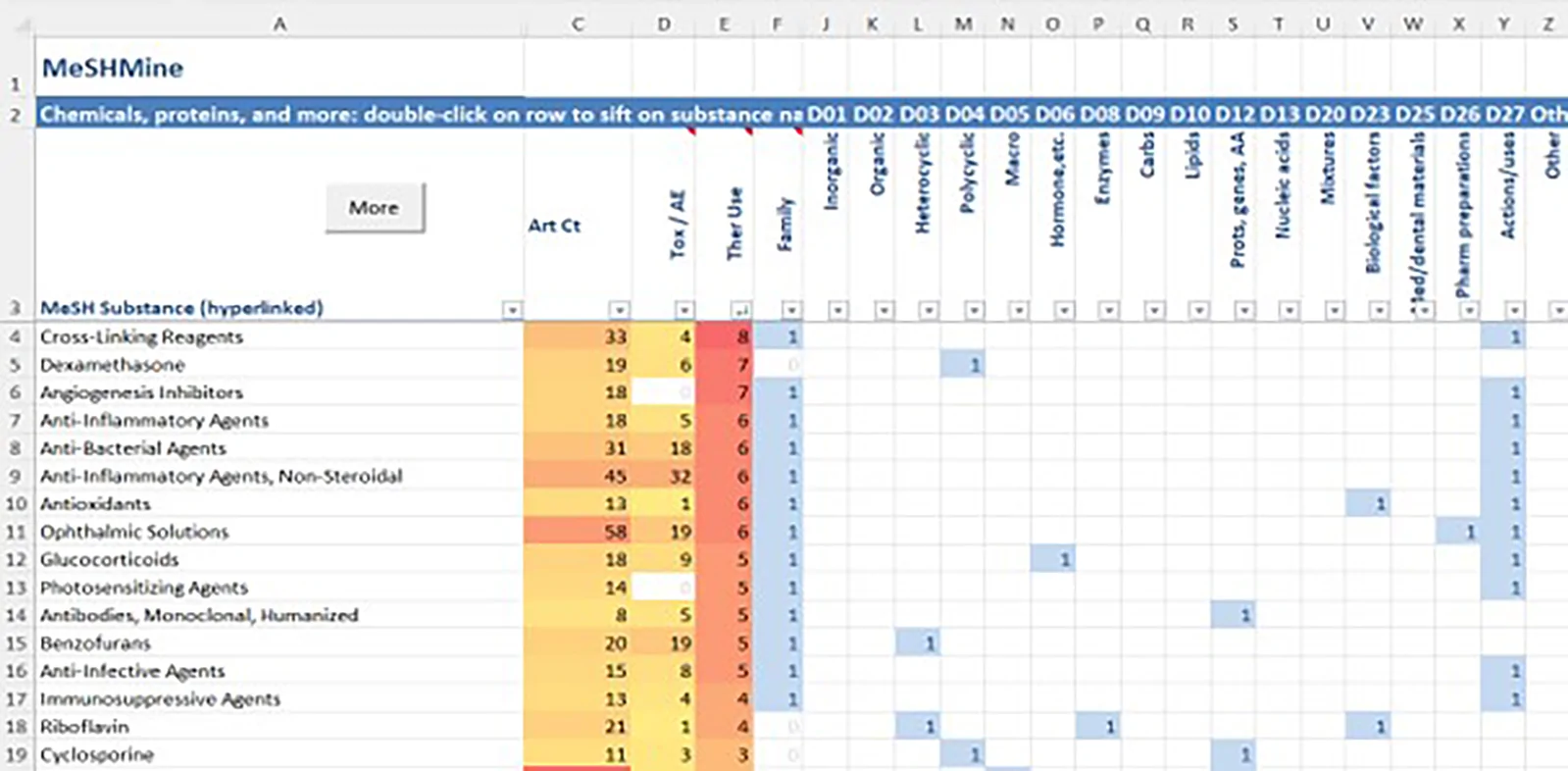
The MeSHMine sheet after running MeSH Mine with the cornea and chemicals query.

MeSH terms are often associated with secondary or qualifier terms that indicate specific aspects of the substance described in a particular article. AS tracks and counts the occurrences of the “drug therapy” qualifier and the combination of “toxicity” and “adverse effects” qualifiers. The counts of the substance annotated with these qualifiers are listed in columns D and E as shown in Figure 7. As a result, researchers interested in drugs associated with corneal treatments can sort on column E listing therapeutic use – *THER USE*, a MeSH qualifier or subheading.

**Figure 8.**
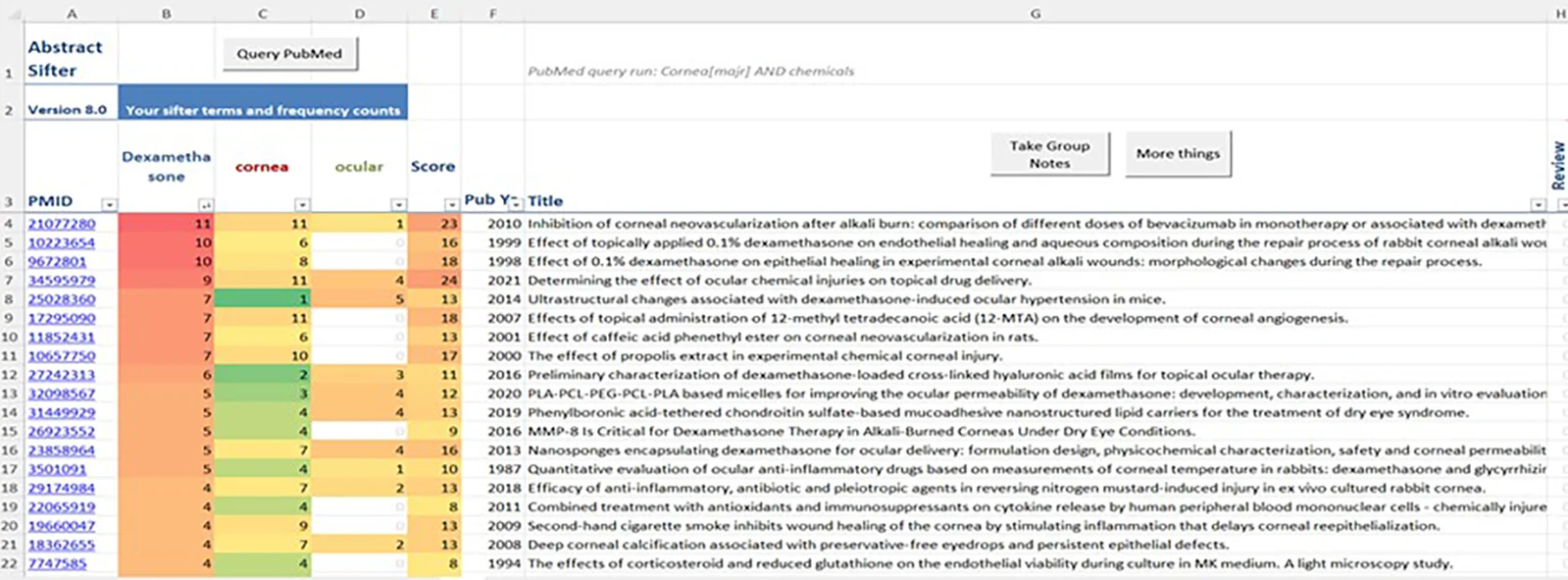
Double-clicking on a row of the MeSHMine sheet writes the corresponding substance to the B3 sifter cell and sorts.

In this example, sorting on therapeutic use sorts many chemical families to the top. The chemical dexamethasone is also sorted to the top and the associated counts show that it is annotated both for its therapeutic use and for its adverse effects. Double-clicking on the Dexamethasone row causes the AS to send the chemical name to sifter cell B3 on the Main sheet and sort by the term counts. The article titles can then be reviewed to see potential roles that the drug may play in corneal diseases ( Figure 8).

Similarly, column D on the MeSHMine sheet can be sorted to see which chemical annotated with toxicity or adverse effects is most annotated with the cornea. Mustard gas is third on the ranked list (not shown) and indeed there is a large literature describing the detrimental effects of this gas on the cornea.

**Figure 9.**
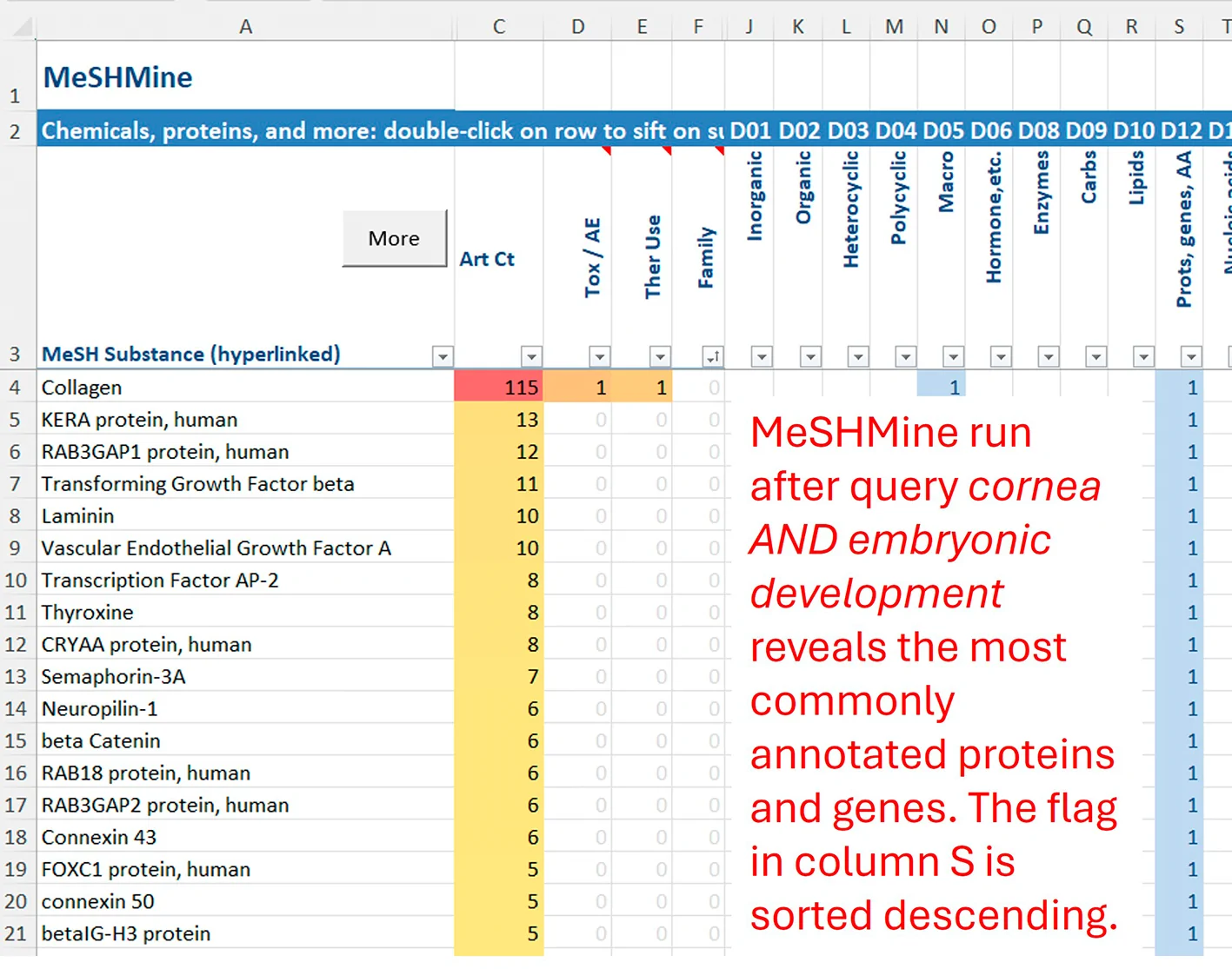
Using MeSHMine to find proteins and genes.

The MeSHMine feature is also a powerful tool to identify the genes and proteins associated with a particular biological process. In the next example shown in Figure 9, the query *cornea AND embryonic development* has been executed, followed by MeSHMine function to identify what proteins and genes are most associated with the development of the cornea. In Figure 9, the MeSHMine sheet is sorted by the protein and gene flag and article count. This action reveals the most annotated proteins and genes in the corpus and can provide important insight into biological pathways. Each protein or gene term is hyperlinked to the MeSH browser.

**Figure 10.**
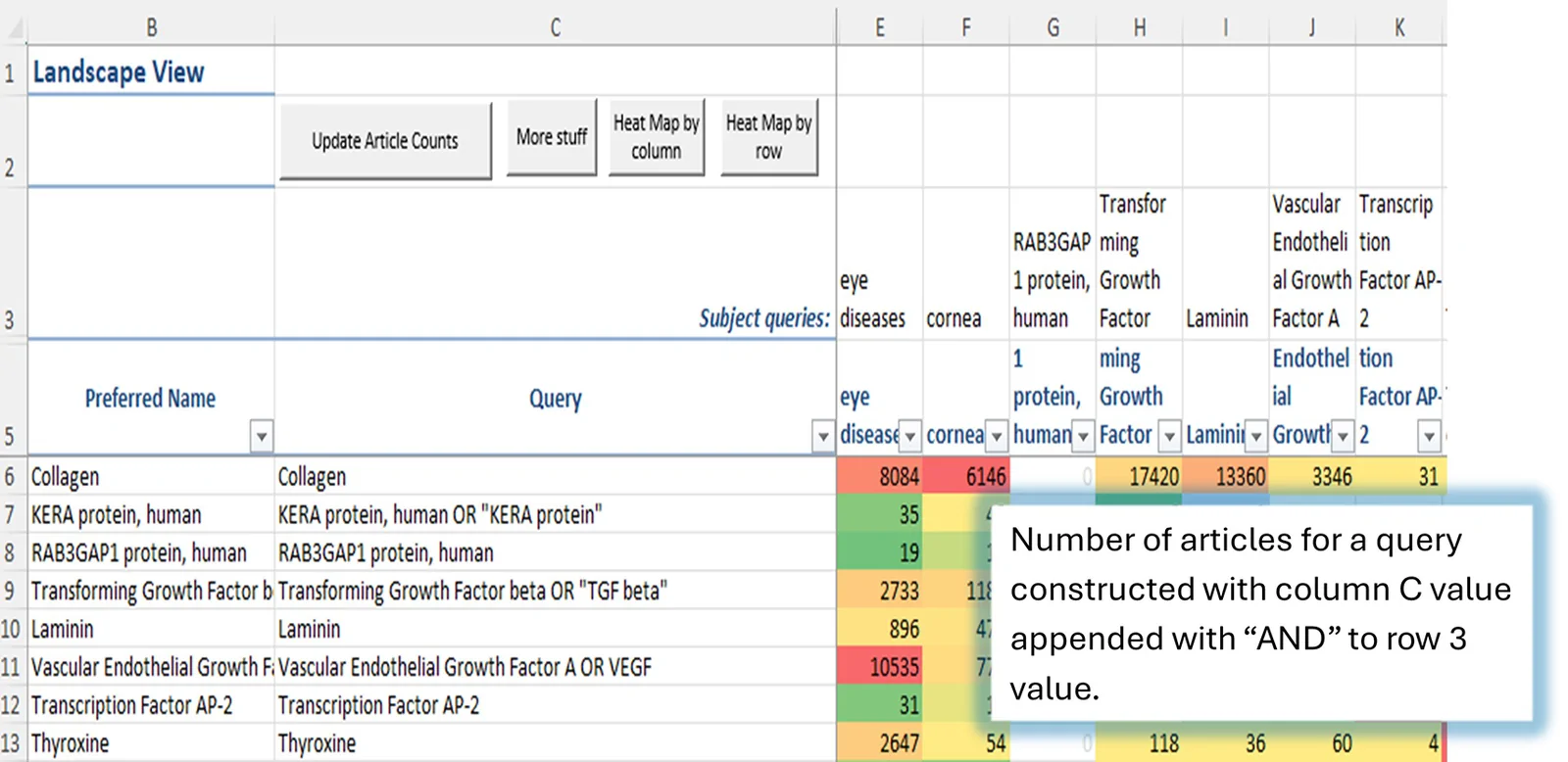
Learn more about the genes and proteins using the Landscape sheet.

MeSH mining can reveal entities that the researcher may want to follow up on. A logical next step to investigate entities is to copy the associated list of names to the Landscape sheet and explore their literature records. Figure 10 lists the retrieved article counts showing the relationship between the entities and cornea as well as between the genes. This information can be valuable in, for example, building gene networks.

*Working with PDFs*

Another way to extract entities and identify them is to retrieve or copy entity names such as chemical names from a PDF, Word document, or Excel file. AS can use the chemical name in an Excel cell, pulled from another source, and look up additional identifiers for those chemicals. Once retrieved, the EPA DTXSID can be used as input into functions that call a large variety of chemical information.

The steps in extracting data from a PDF file and retrieving information for the chemicals are illustrated in the next figures.

**Figure 11.**
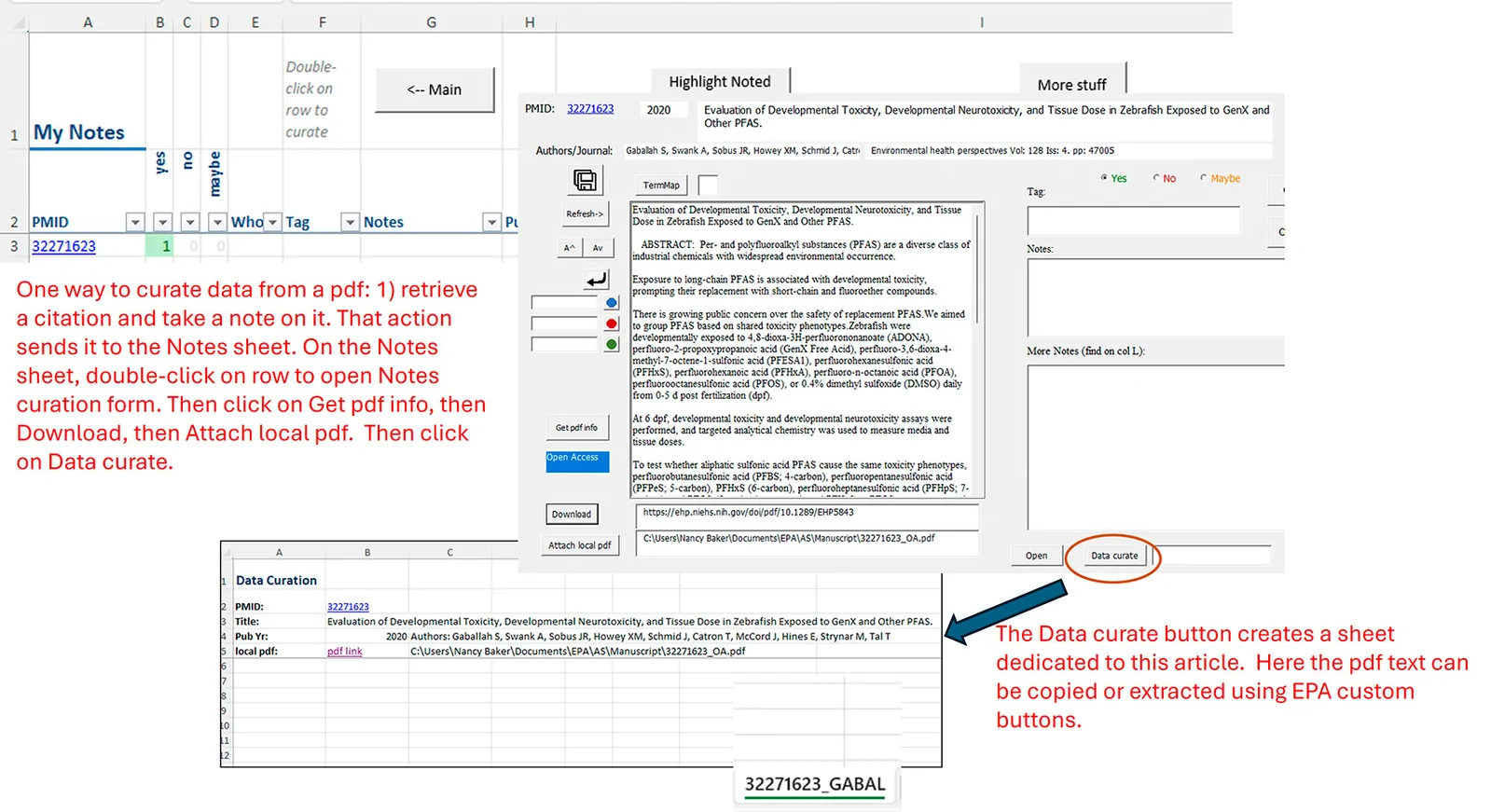
Steps in creating a curation sheet.

Consider the example article: https://pubmed.ncbi.nlm.nih.gov/32271623/. Using AS to query PubMed, the article can be retrieved with a PubMed ID of 32271623[uid] as the query. The record is inserted into the Notes sheet and double clicking on the row brings up the record in the Notes curation form. In order to see whether the article pdf is open access, and to retrieve it if it is, the user can click on “Get pdf info” to Download. To create an Excel worksheet for the data in the PDF, the user clicks on “Data curate”. A new worksheet is inserted with a label constructed from the PubMed ID and the first author. These steps are illustrated in Figure 11.

**Figure 12.**
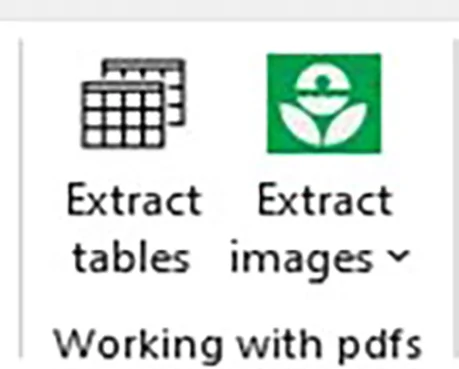
Working with PDFs group on EPA Custom ribbon.

In this example, the 32271623_GABAL sheet is where the user can copy and paste any data or text related to this article. Alternatively, the user can tell AS to extract tables and figures from the PDF. To use this AS function, the user clicks on Extract tables or Extract Image (see Figure 12) on the EPA custom ribbon. This example shows how to extract a table from a pdf where the publication contains a list of chemicals for which unique DTXSID identifiers can be extracted for the chemicals and then used to obtain other associated chemical data.

Clicking on the *Extract tables* button results on the custom ribbon tells the AS to look for the pdf with the hyperlink in cell B5 and open it using Microsoft Word. As Word opens the PDF, it converts text in tables into table objects. The AS reads the rows and columns of the table objects and writes the results to the curation sheet.

**Figure 13.**
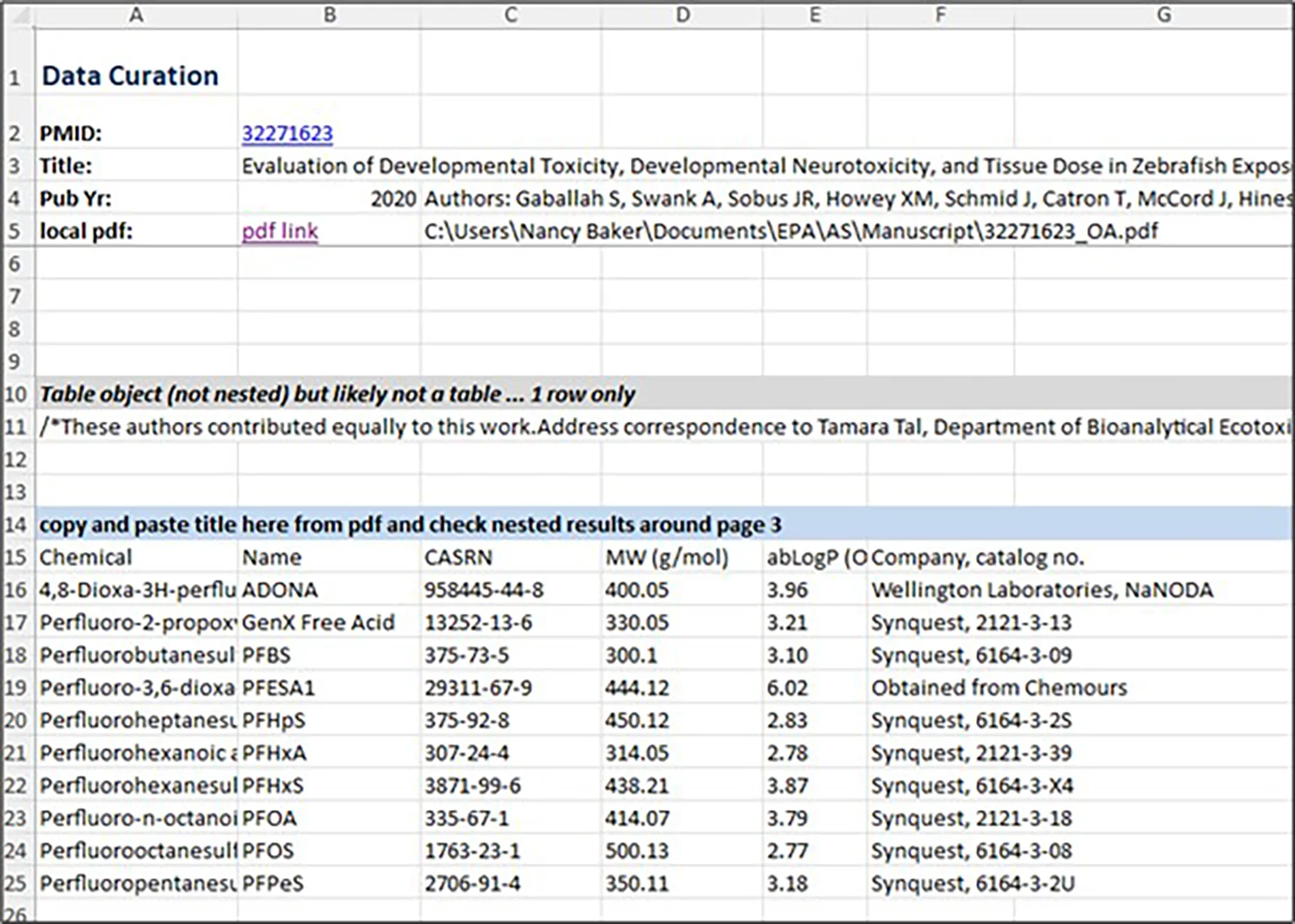
Data extracted from the PDF.

It should be noted that tables embedded in PDFs can vary significantly in how they are formatted and Microsoft Word cannot always accurately convert complex tables into Excel rows and columns. Formatting characters are sometimes missed, leading to concatenated data. As a result, the extracted data in the curation sheet should be reviewed by manual inspection and compared to the PDF to ensure accurate conversion. In this example, the PDF is well formed, and the extracted data shown in Figure 13 are accurate.

*Chemical entity identification*

**Figure 14.**
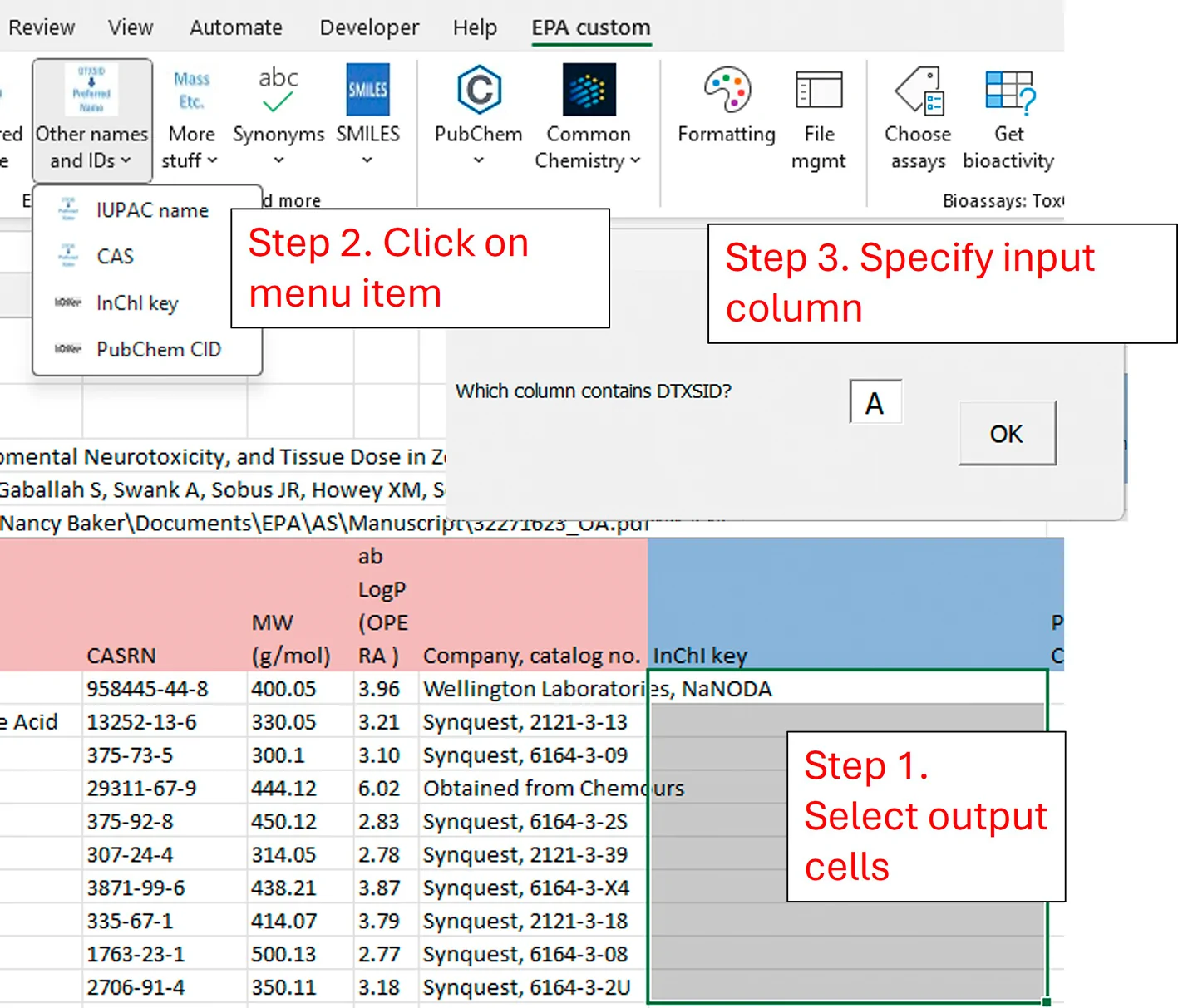
Basic steps in retrieving chemical data using functions on the EPA custom ribbon.

The data extracted from the PDF in the previous section has chemical names and CAS registry numbers but lacks the EPA DTXSIDs that will link them to chemical data such as InChI keys and SMILES. The process of retrieving DTXSIDs is controlled through functions available through the EPA custom ribbon ( Figure 1). The functions available through the EPA ribbon work in a similar fashion: the user selects the cells for the output, clicks on the menu selection for the function, and then enters the column with the corresponding input value. The input values used to retrieve the DTXSIDs are generally chemical names or CAS registry numbers. Figure 14 shows the three steps associated with running the ribbon functions.

The functions that retrieve chemical information and place it in the specified cells are calling the EPA’s CTX-APIs. ^2^ These APIs retrieve from the DSSTox database, the same database used in the CCTE Dashboard. ^9, 10^

Chemical entity identification is important because the chemical identifier gives the AS fast and accurate access to other data specific to that chemical, like properties and structural information. The AS makes the process of moving from chemical names to a structured dataset easier.

**Figure 15.**
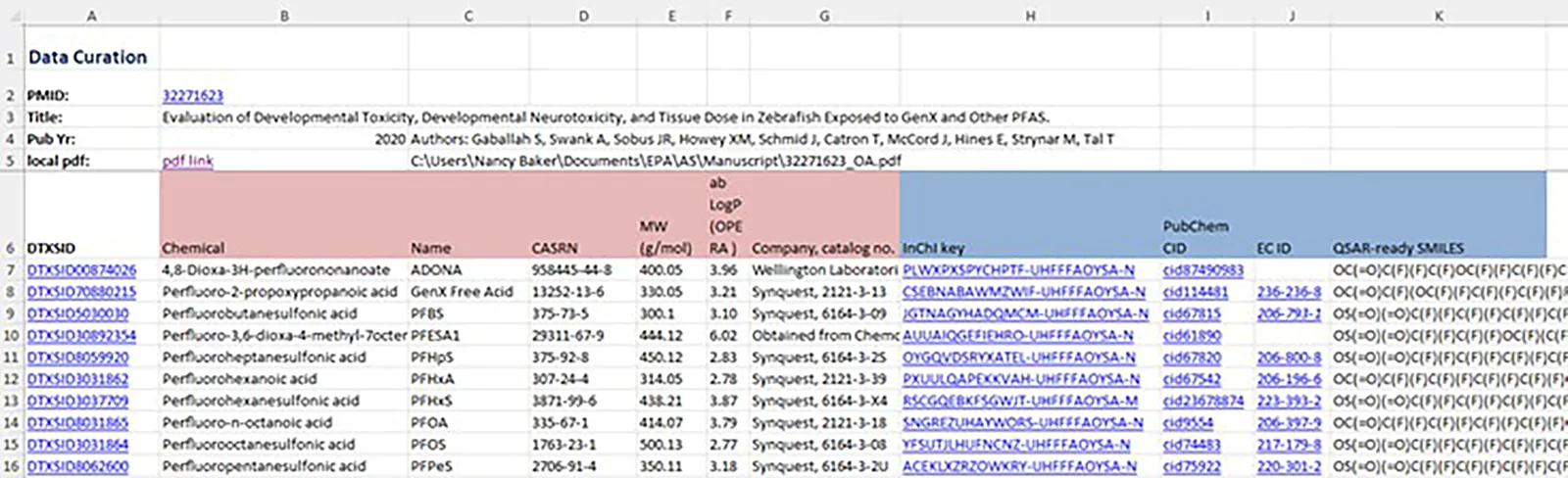
Sample of the types of data that can be retrieved using the EPA custom ribbon functions.

Figure 15, for example, shows how a simple list of chemical names can be enhanced with chemical data from the DSSTox database once the DTXSID (column A) has been retrieved. The columns with the blue headers on the right have been retrieved through the AS functions. The example includes structural data and external identifiers with hyperlinks. The User Guide and available training videos walk through the steps in greater detail.

**Figure 16.**
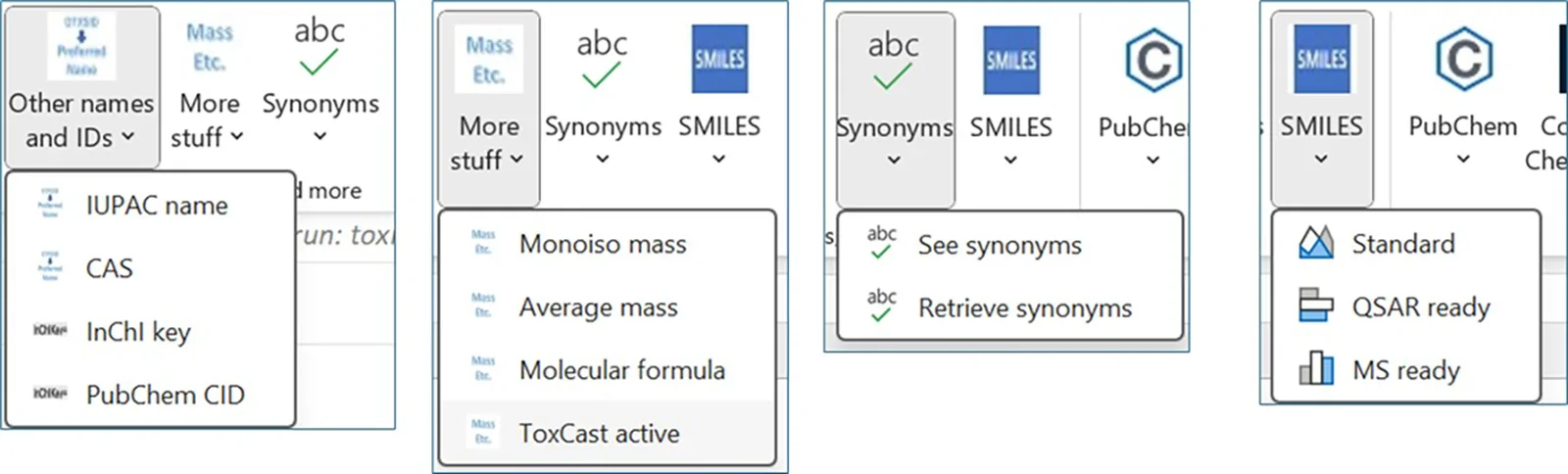
Function menus for selected ribbon groups in the EPA custom ribbon.

Figure 16 shows the variety of data accessible from DSSTox database via the CTX-APIs.

**Figure 17.**
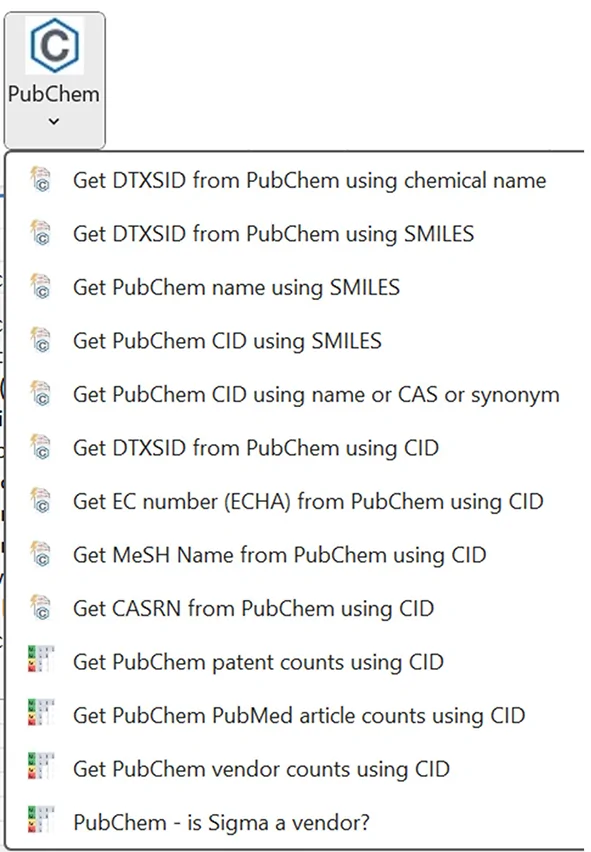
PubChem functions on the EPA custom ribbon.

AS also retrieves data from PubChem, the chemical database developed by the National Institute of Health. ^6, 7^ Figure 17 shows the list of PubChem functions with input values. A third source of information is Common Chemistry, a database made available by Chemical Abstracts Service (part of the American Chemical Society) accessed through their API. ^11^

### EPA chemical lists

**Figure 18.**
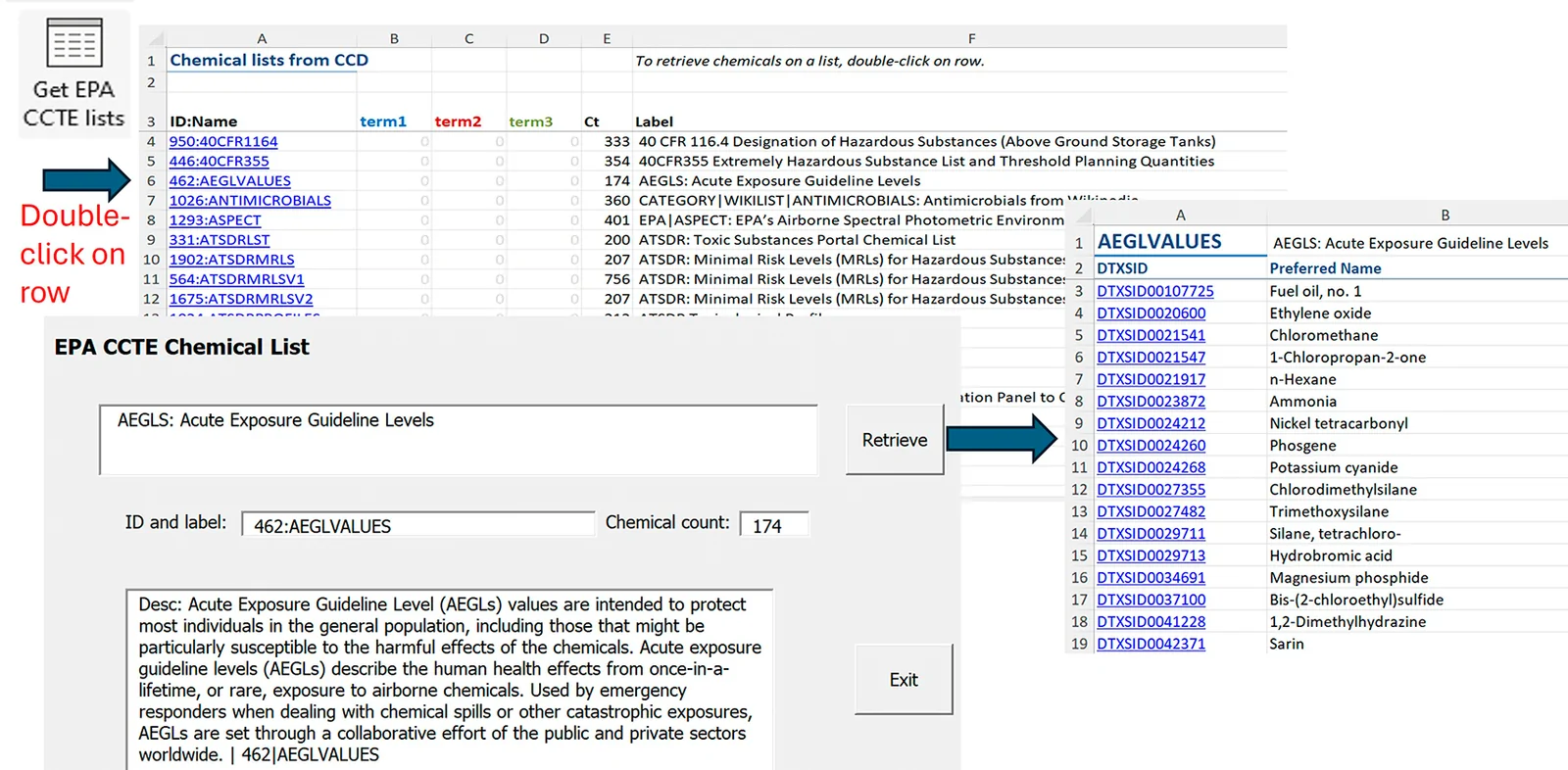
Steps in retrieving chemical list of lists from the EPA custom ribbon and then the chemicals from a selected list.

Another important feature of AS is the DSSTox chemical list retrieval functionality. This feature is a direct way to import sets of chemicals into Excel and produces results very similar to the chemical list retrieval functionality on the CCTE Dashboard. The EPA has made over 500 chemical lists available to the public. These include chemicals tested by the EPA (e.g., https://comptox.epa.gov/dashboard/chemical-lists/TOXCAST), lists of chemicals by use (e.g., https://comptox.epa.gov/dashboard/chemical-lists/SWISSPEST), and lists of chemicals based on structural moieties (e.g., https://comptox.epa.gov/dashboard/chemical-lists/PFASOECD). On the CCTE Dashboard, the chemicals in a selected list can be downloaded with selected additional data fields. In AS, the list of chemical lists can be retrieved by clicking on the icon in the EPA Custom ribbon. From there, the list can be searched and sorted, and a single list can be retrieved by double-clicking the name. Each chemical comes with the DTXSID identifier, making retrieval of additional data (e.g., SMILES or structure diagram) straightforward. This sequence of events is illustrated in Figure 18.

### More ways to get chemicals from publications

Many research groups, including the EPA, extract chemical names from publications, associate those chemical names with unique identifiers, and make them available to other researchers through various means, including as downloadable datasets and via APIs. Table 1 summarizes some of the projects that extract and identify chemicals from scientific publications and make them available to users. It is worth noting that many of these projects deliver more information than just chemical substances, but for the purposes of this publication the focus is chemical entities.

**Table 1.** Projects that extract and identify chemical entities from publications and make them accessible through API calls.

Project	Organization	Chemical identifier	Corpora	Coverage	Method	Available in AS v8?	Reference
PubMed MeSH term annotation	NLM NCBI	MeSH substance identifier	PubMed	37 million PubMed/Medline records; estimated 15 million citations have chemicals	Historically - expert curated; Currently - automated text mining	Yes	^4, 5^
PubChem	NLM NCBI	PubChem substance and compound identifiers	PubMed	Unknown	Automated text-mining; some deposition	Yes, by article	^6, 7^
Chemfindable	EPA CCTE	DTXSID	PubMed, Methods documents, Fact sheets	In progress	Expert curation, some automated identification	Only for PubMed publications. More planned for AS v9	
PubTator	NLM NCBI	MeSH identifier	PubMed abstracts; PMC full text	35 million abstracts; 6 million full text articles	Automated text-mining	No, planned for AS v9	^12^
Europe PMC	EMBL EBI	ChEBI ID	PubMed; some PubAg, dissertations, preprints	45 million abstracts and publications	Various. See https://europepmc.org/Annotations	No, planned for AS v9	^13^
Comparative Toxicogenomics Database	NC State	MeSH substance uid	PubMed	>149,000 PubMed articles	Expert curated	No, planned for AS v9	^14^

**Figure 19.**
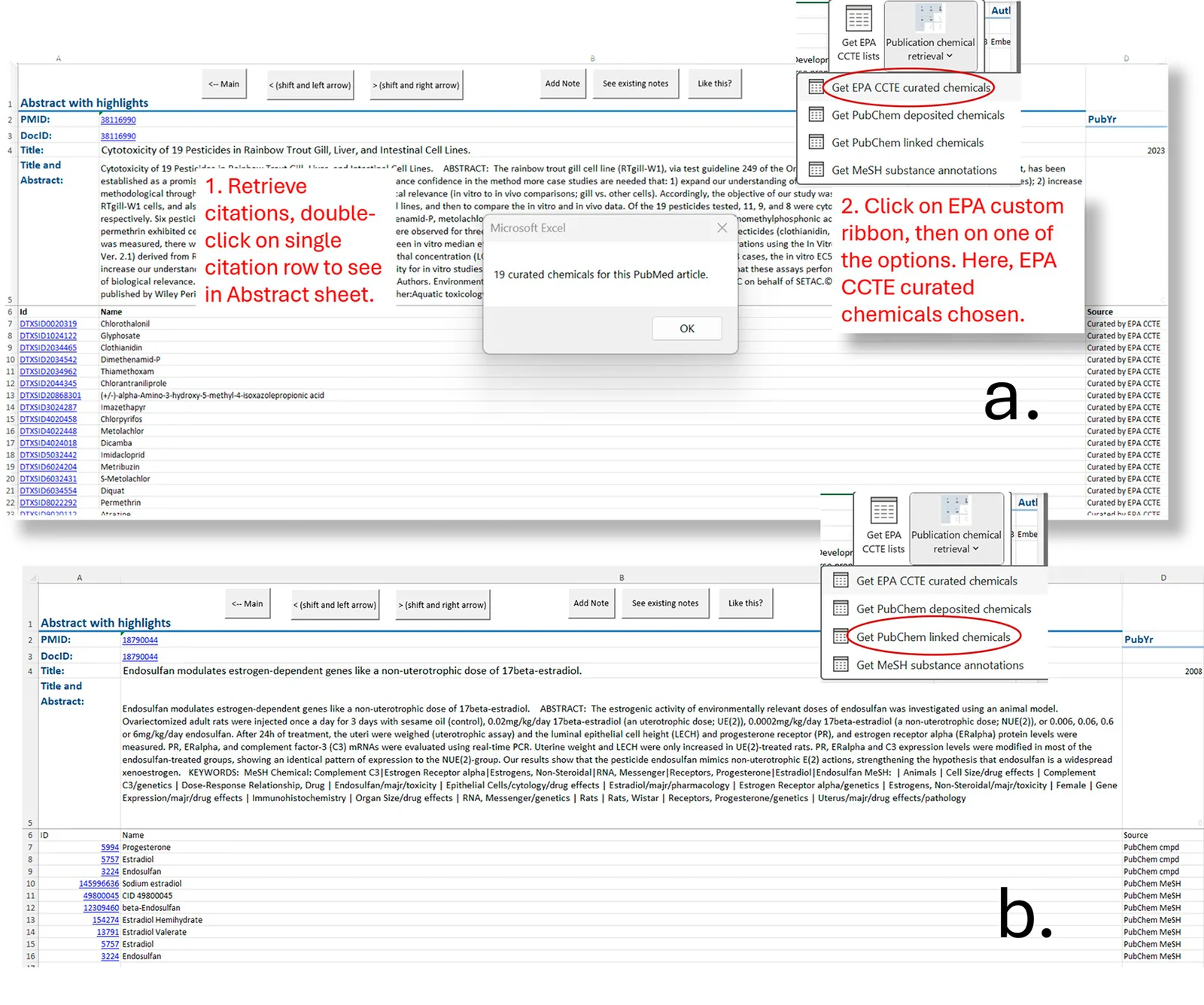
a and b. On the Abstract sheet how to retrieve chemicals associated with the article. (a) shows chemicals curated by EPA. (b) shows text-mined chemicals from PubChem.

AS version 8 delivers the chemical entities from several of the projects listed in Table 1. Using the MeSH Mine feature, all chemical or substance MeSH terms are extracted from the corpus of citations on each project’s Main sheet, collected, counted, and written to the MeSHMine sheet with hyperlinks to the MeSH Browser and double-click navigation to the citations on the Main sheet. In contrast, access to chemicals associated with PubChem and the DSSTox database is performed at the single article level. Figure 19a and 19b indicate how this functionality works. With a single article loaded into the Abstract sheet and using the Chemical Lists section of the EPA custom ribbon, DSSTox curated chemicals are accessed by selecting “Get curated EPA DSSTox chemicals”. This retrieved a total of 19 chemicals for the selected article. The number of publications that have associated chemicals that can be retrieved by end users is growing but still under 1000. Figure 19b shows retrieval of PubChem linked chemicals.

### Understanding sets of chemicals

While the last section reviewed the approach to extract chemicals from publications and how to download existing lists of chemicals from the EPA DSSTox database through CTX-API calls, this section reviews approaches that provide insight into sets of chemicals, whatever their origin. The AS provides insight into biological activity of chemicals through ToxCast and Tox21 assay data ^15– 17^ and insight into chemical structure through ToxPrint data ^18^ and chemical structure diagrams. The bioassay and ToxPrint data are displayed in a matrix format with one chemical per row and the associated data in columns. This matrix format lets the AS take advantage of similarity metrics and column sorting to make the data more accessible.

*Bioassay hit calls*

**Figure 20.**
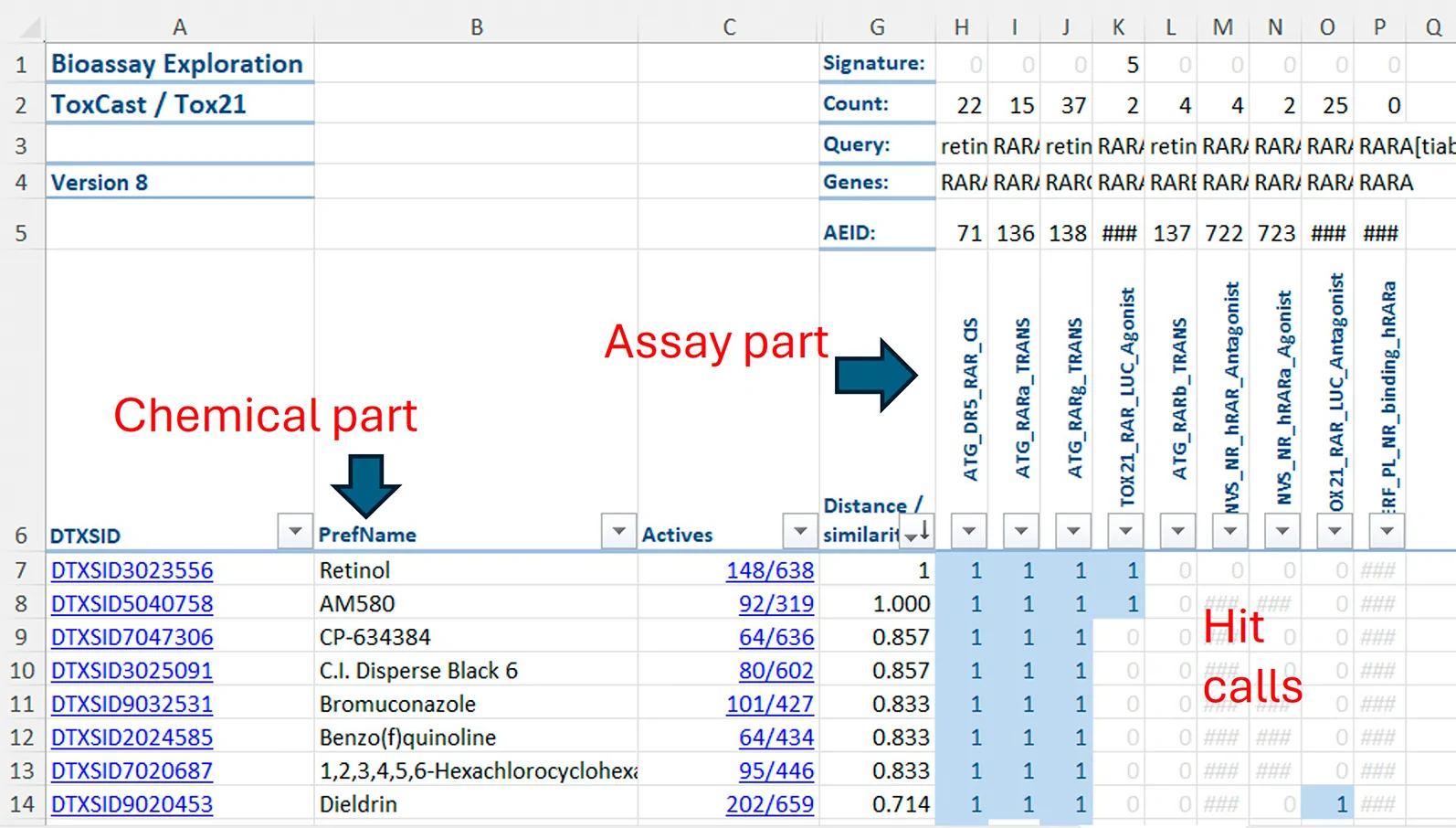
Sections of the Bioassay sheet.

**Figure 21.**
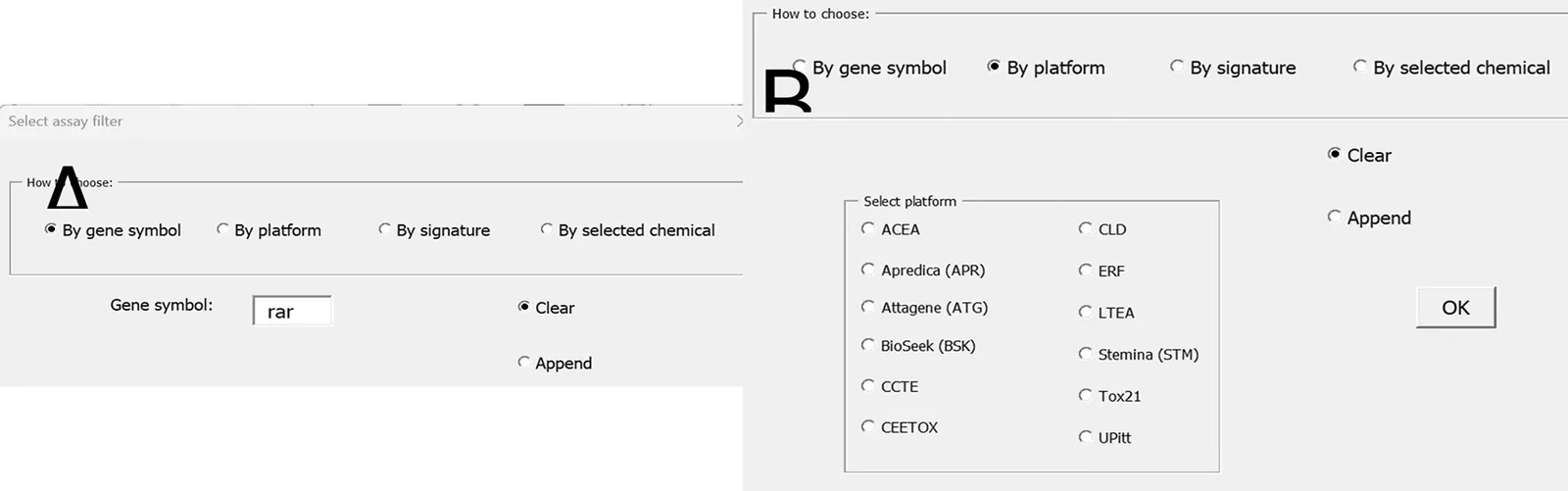
There are four ways to select assays. Shown here is how to select by gene symbol (A) and by assay platform (B).

A subset of the Bioassay sheet is displayed in Figure 20. Users can build sheets by providing the chemicals (with DTXSIDs) in column A, then specifying which assays to retrieve hit calls for ( Figure 21). More detailed guidance is provided in the user guide.

The matrix format of the Bioassay sheet can be challenging to interpret, but the AS provides functions to ameliorate these challenges. For example, if the hit call portion of the sheet is large and hit calls are sparse, it is difficult to view a chemical’s results. Therefore, the AS Bioassay button menus allow for sorting by a selected chemical, an option that sorts the columns to order all assays with positive hit calls to the left of the sheet where they can be easily viewed together. The chemical used to perform sorting on is inserted in row 7 below the header rows, shown in Figure 20 as the chemical Retinol. After sorting, Retinol’s positive hit calls in this set of assays are easy to view. This figure also shows that the similarity function has been run (also available in the Bioassay section of the ribbon) and below Retinol are the chemicals in this set that have bioassay hits similar to Retinol. More detail regarding how this similarity metric is calculated can be found in the user guide.

**Figure 22.**
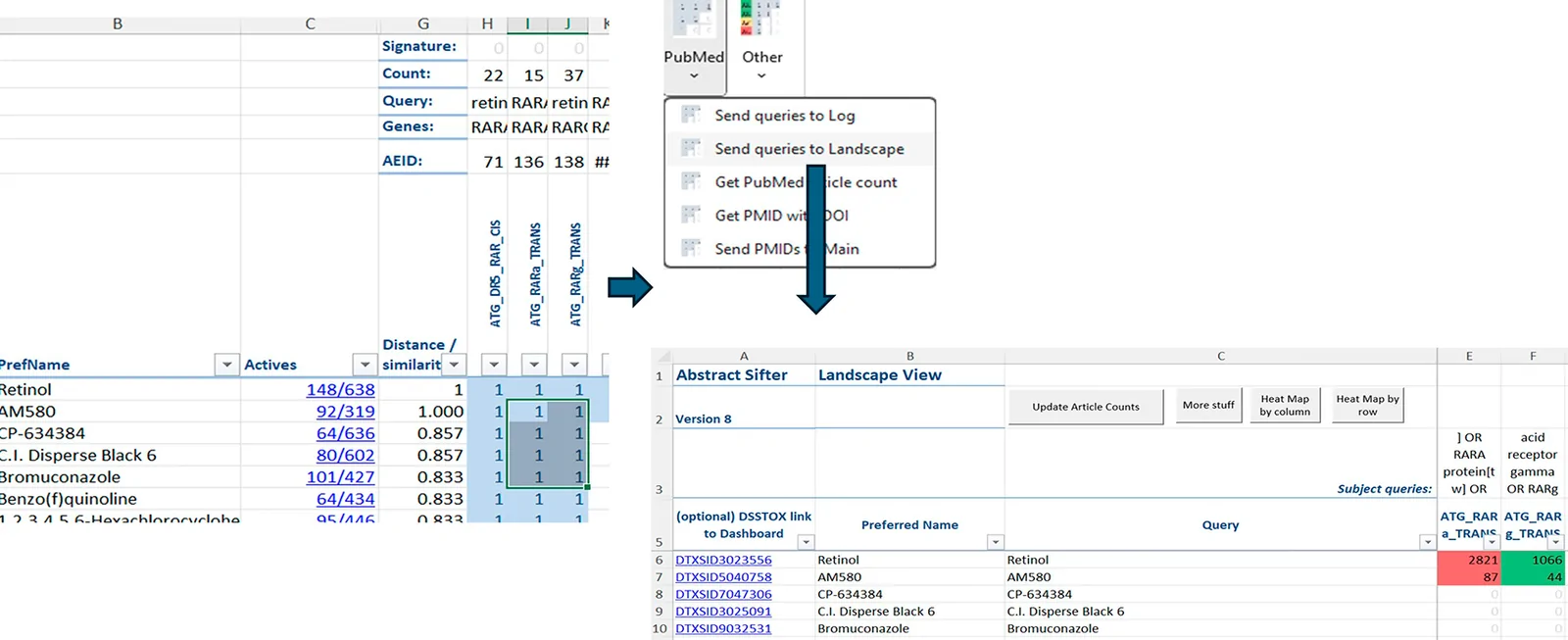
Through the menu option shown, the chemicals and genes associated with the assays are written to the Landscape sheet where queries can be run to visualize article counts and run the queries.

AS is primarily a literature tool, so even the Bioassay sheet provides a way to query PubMed. When links between a chemical and a gene are indicated by a positive hit call – or not indicated by a positive hit call – queries to PubMed can be conducted quickly to support or check the bioassay evidence. Double-clicking on any cell in the hit call region causes AS to construct a query from the chemical name and the genes associated with the assay. This query is placed in the main query form, ready to be executed, and the results are sent to the Main sheet for browsing. Using another menu option on the EPA Custom ribbon, the chemicals and genes can be sent to the Landscape sheet ( Figure 22). The user can then get an overview of the literature relationship between the chemicals and genes and navigate to the relationships of interest.

*ToxPrints*

The second matrix formatted set of functions is accessible through the ToxPrints sheet. ToxPrints are a set of 729 structural fragments that can be used for structure-based analyses. ^18^ For all chemicals in DSSTox, ToxPrints have been pre-computed and the EPA CTX-APIs can retrieve and deliver these ToxPrints to AS. In version 8 of AS, a sheet called ToxPrints has been added to house the retrieved data. The user inputs DTXSID chemical identifiers into column A, then by clicking on the EPA custom menu option, retrieves the ToxPrints. The data are binary: 1 = yes, the chemical has a specific chemical ToxPrint; 0 = the chemical does not have the ToxPrint.

For a set of chemicals, the ToxPrint data matrix can be very sparse – with 729 possible ToxPrints a chemical will generally only contain a small number. Similar to the Bioactivity sheet, the ToxPrint sheet has functionality that sorts the columns by a selected chemical so that the user can inspect the Toxprints associated with that chemical without scrolling interminably to the right. Comparing one chemical to the others is possible visually by viewing colorized patterns of 0s and 1s, but AS can also calculate the Tanimoto distance between the row 6 chemical and the rest of the set. The sorting and Tanimoto calculation are functions available through the EPA custom ribbon.

**Figure 23.**
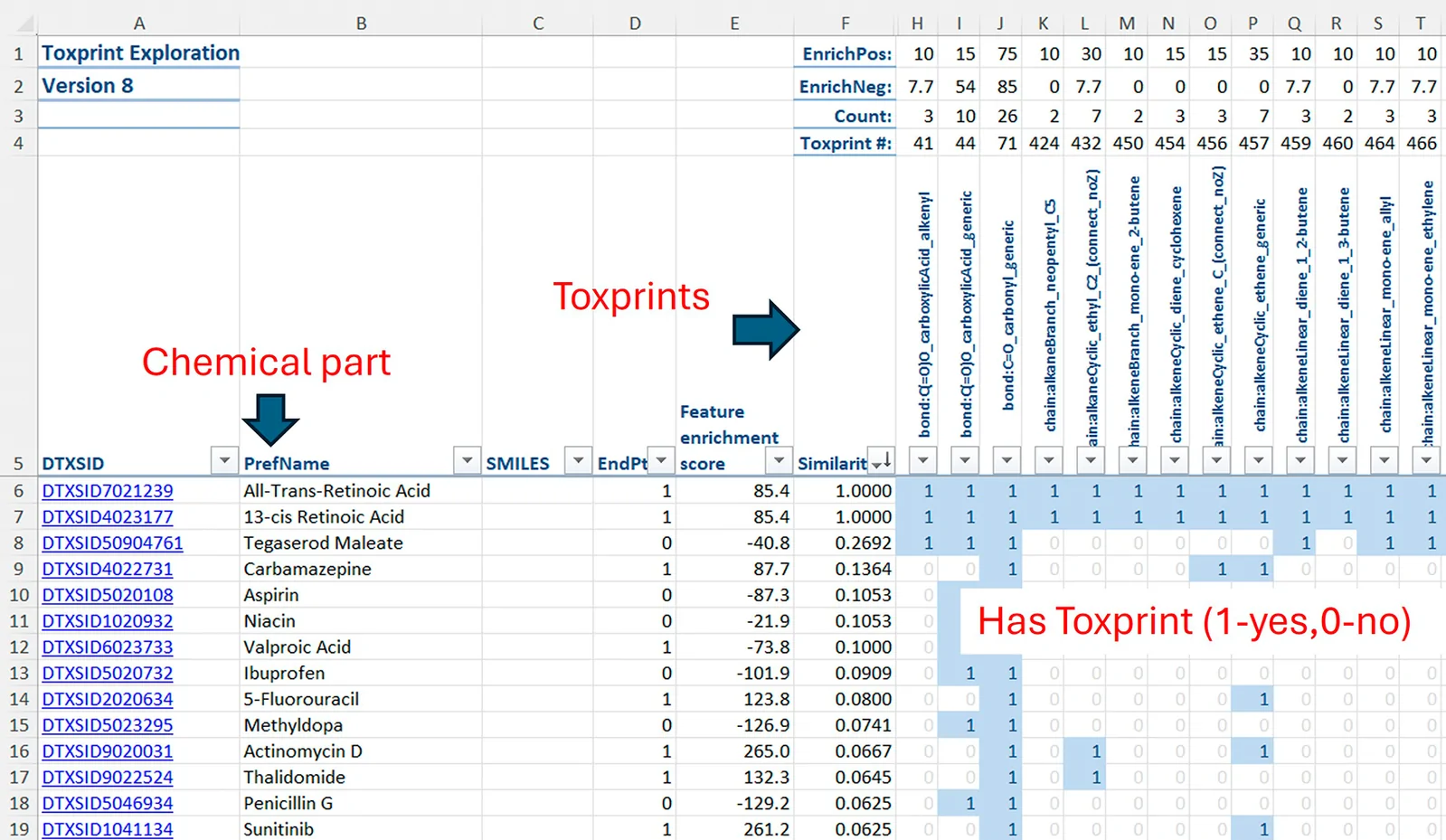
Populated Toxprint sheet with sections shown. Similarity metrics from the Row 6 chemical have been calculated.

Figure 23 shows a fully populated ToxPrint sheet. In this example the ToxPrint columns are sorted for All-Trans-Retinoic acid and the Tanimoto similarity of all the chemicals compared to All-Trans-Retinoic acid has been calculated and placed in column F. Additional functionality includes methods to calculate the enrichments of ToxPrints associated with binary endpoints. Interested readers will find this functionality described in the user guide.

*Chemical structure diagrams*

**Figure 24.**
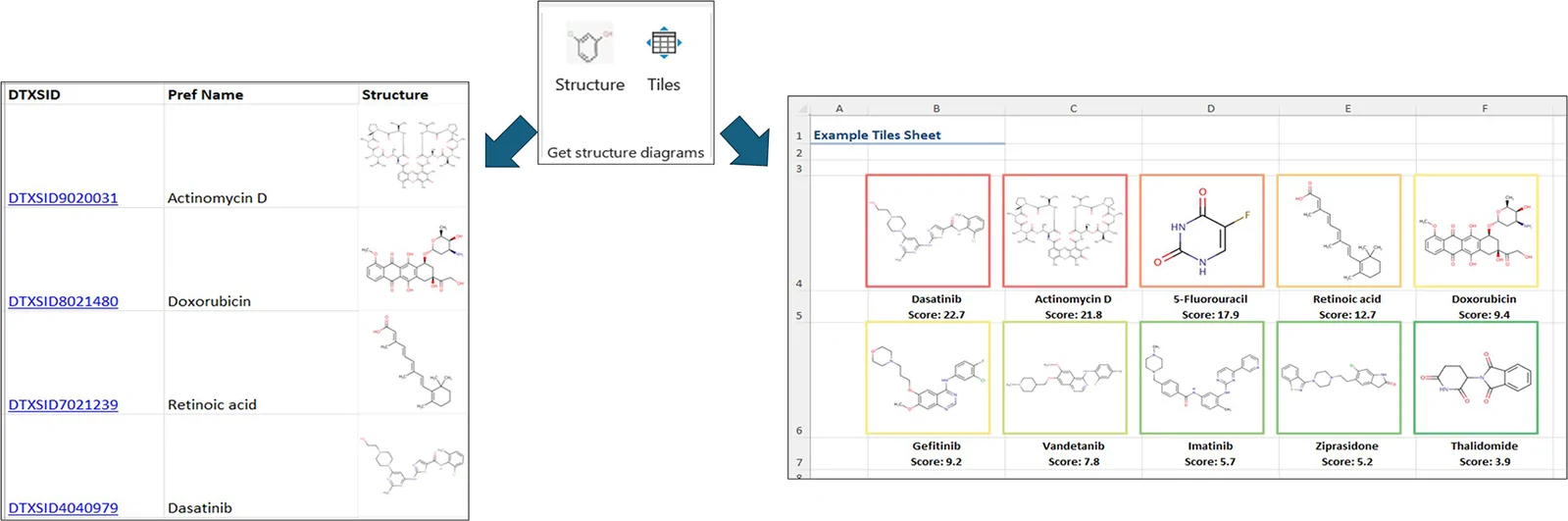
Two ways to retrieve chemical structural diagrams.

Chemical structure diagrams are an important way to visualize chemicals. AS version 8 has two ways to retrieve structure diagrams, both accessible from the ribbon group shown in Figure 24. The first method works like other chemical data retrieval and inserts the structure figure in a cell associated with the DTXSID in the same row. The Tiles option creates a new sheet called Tiles and on it arranges the chemical diagrams in rows of five, a format that makes visual comparison easier and is more suited for presentations or publications. Additional options include adding chemical names and additional data and coloring the tile borders. The User Guide should be consulted for a step-by-step description of this functionality.

## Discussion

New research in a particular scientific domain rests on effective use of previous work. The Abstract Sifter tool handles the challenges of finding relevant information in the 37 million citations encompassed by PubMed. AS contributes to advancing new research with increased efficiency and better results.

Using AS, a large complex corpora of retrieved PubMed citations can be built. With term-mapping, the size of the corpora is not a barrier to finding relevant citations quickly and ranking the citations with high accuracy and confidence. The rankings are transparent and reproducible. The dynamic nature of querying, sifting, and term-mapping means that the researcher can work through ideas and hypotheses without being penalized by lost time. The recording of queries on the Log sheet lets a researcher keep track of this exploration and provides a convenient way to rerun earlier queries. Note-taking functionality helps researchers keep track of their thoughts, opinions, intentions, and screening decisions.

While AS is a powerful tool for research in any biomedical domain, AS version 8 has been enhanced to make it more valuable for chemical research. In the example discussed above, the MeSHMine functionality lets the researcher find the chemical substances linked to a given disease. Chemicals linked causally to the disease, treating the disease, and the genes and proteins playing a role in disease mechanisms can all be mined and displayed with counts and navigation shortcuts. Exploring the chemical substances linked to a disease is an effective introduction to a disease state and the evidence accumulated by previous researchers. These literature mining methods and concepts are translatable to other research problems.

The researcher who builds on prior work will often need to take the chemicals from a publication and include them in future work. Version 8 of Abstract Sifter makes chemical name extraction much easier and the functions that call the CTX-APIs make it possible to assign the unique DTXSIDs to chemical names. Version 8 of AS streamlines the entire process of finding past studies of chemicals and building sets of chemicals for new studies.

Chemicals are often analyzed in sets. Studying them in sets is a powerful methodology, because, as long as a unique identifier like the DTXSID is established, many external data points such as structural features can be brought to bear in the search for patterns, relationships, cause and effect, and ultimately understanding. AS is a useful tool for building chemical sets. Chemicals used in publications can be extracted and matched with their unique DTXSIDs and more data points retrieved. If the chemicals are in ToxCast or Tox21, their results in the assays can be evaluated and compared visually or mathematically through distance metrics. Structural diversity of a set of chemicals can be investigated through the ToxPrints functionality or through the innovative ways to visualize chemical structures. Using the Landscape sheet, published effects of chemicals can be visualized and navigated.

There is an irony in the trend to study chemicals in sets. In a publication, the more chemicals studied, the less likely the names of the chemicals will be in the title, abstract, or keywords of the publication, the sections of the publication available to a search engine. Chemical lists will more likely be in the text or tables in the body of the PDF or in supplemental information. Except for PubMed Central (PMC) articles, the PDF and supplemental information are outside the reach of a search engine and therefore the chemicals are not findable. This means that a researcher aiming to assemble data about a certain chemical could miss important publications and information about that chemical. While the researchers who study chemicals increasingly adopt methods that let them study more than one chemical at a time, the less useful this information is: if it can’t be found, it can’t be used.

The efforts of NLM, European PMC, and the EPA to curate, extract, and store chemical entities from publications represent valuable progress in resolving this issue. AS and its related EPA projects will continue to increase the size of its curation database and the ways to deliver chemical information.

The AS depends on the platform and resources it is built upon. Microsoft Excel is a powerful tool on its own and, as the foundation for AS, the user gets strong data-handling capabilities in addition to a familiar and intuitive user experience. Microsoft regularly improves and extends Excel. The VBA programming language makes the delivery and navigation of information to the Excel front end possible, and the contributions of a community of programmers ease the technical challenges. The ability to use API calls to rich databases and bring data back to the familiar Excel sheets is possible because of the vision and work of governmental organizations like NLM and the EPA.

As with version 8, future versions of AS will streamline literature tasks. Plans for future development include retrieval from repositories other than PubMed including European PubMed Central and technical documents. The tool will continue to be applied to EPA projects to find innovative ways to extract and deliver chemical information. The Abstract Sifter draws on rich resources to make a powerful tool that can accelerate the pace and thoroughness of research, particularly in the chemical domain.

## Disclosures

This paper does not necessarily represent the views or policies of the U.S. Environmental Protection Agency.

## Software availability

Abstract Sifter version 8 available at: The Abstract Sifter tool, user guide, and any future updates: https://www.parlezchem.com/abstract-sifter/

Software source code available at https://doi.org/10.5281/zenodo.20312161

Software licensed under Creative Commons Attribution 4.0 International.
