# Supplementary material for: Abstract Sifter version 8: Focus on the chemical literature
Source: F1000Res. 2026 Jun 6;14:Chem Inf Sci-260. Originally published 2025 Mar 4. [Version 2] doi: 10.12688/f1000research.160617.2 (PMC13554733; doi:10.12688/f1000research.160617.2)
Supplement: Peer review history

# Reviewer report: Reviewer response for version 2

**DOI:** 10.5256/f1000research.202196.r500804

**Published:** 2026-07-28

**Authors:** Holli-Joi Martin

**Affiliations:** 1 University of North Carolina Chapel Hill, Chapel Hill, NC, USA

Since the original Abstract Sifter was introduced in 2017, the tool has grown from a way to search and review scientific papers into a more complete system for working with chemical literature. Version 8 adds new features that can pull chemical names from papers and tables, connect them to EPA chemical identifiers, and retrieve related toxicity and exposure data. These updates make it easier for researchers to move from finding relevant studies to organizing and using chemical information.

 The main text is well formatted and easy to follow. The many simple figures are appreciated. It is my opinion however that the discussion could be less repetitive and the authors should more directly address the platform’s limitations. These include its dependence on PubMed indexing, limited access to full text and supplemental files, potential errors in chemical extraction and DTXSID mapping, and reliance on Excel and VBA. The discussion should also better distinguish the novel features of version 8 from existing resources and reduce repetition around chemical-set construction and data retrieval to create more space for validation, limitations, and the settings where Abstract Sifter offers the greatest benefit.

Are the conclusions about the tool and its performance adequately supported by the findings presented in the article?

Yes

Is the rationale for developing the new software tool clearly explained?

Yes

Is the description of the software tool technically sound?

Yes

Are sufficient details of the code, methods and analysis (if applicable) provided to allow replication of the software development and its use by others?

Yes

Is sufficient information provided to allow interpretation of the expected output datasets and any results generated using the tool?

Yes

Reviewer Expertise:

Data curation, data mining, data science, machine learning

I confirm that I have read this submission and believe that I have an appropriate level of expertise to confirm that it is of an acceptable scientific standard.

# Reviewer report: Reviewer response for version 2

**DOI:** 10.5256/f1000research.202196.r491368

**Published:** 2026-06-25

**Authors:** Hunter N B Moseley

**Affiliations:** 1 University of Kentucky, Lexington, USA

The authors have reasonably addressed the issues raised by this reviewer.  Moreover, they have persevered in revising the software and the manuscript under difficult work conditions.

Are the conclusions about the tool and its performance adequately supported by the findings presented in the article?

Partly

Is the rationale for developing the new software tool clearly explained?

Yes

Is the description of the software tool technically sound?

Partly

Are sufficient details of the code, methods and analysis (if applicable) provided to allow replication of the software development and its use by others?

Partly

Is sufficient information provided to allow interpretation of the expected output datasets and any results generated using the tool?

Yes

Reviewer Expertise:

scientific programming; bioinformatics, cheminformatics; systems biology

I confirm that I have read this submission and believe that I have an appropriate level of expertise to confirm that it is of an acceptable scientific standard.

# Reviewer report: Reviewer response for version 1

**DOI:** 10.5256/f1000research.176540.r407559

**Published:** 2025-09-02

**Authors:** Hunter N B Moseley

**Affiliations:** 1 University of Kentucky, Lexington, USA

This manuscript presents a new version of the Abstract Sifter VBA macro-based Excel workbook for pulling chemical information from PubMed abstracts.  The manuscript presents the functionality provided by the software in a fairly descriptive and organized manner.  However, there are significant issues with enabling the software to run in a trusted environment.  

 Major Issues:

 1) Given the limitations of running macros in Excel based on “Trust Center” settings, the authors should mention the requirements and steps needed for Abstract Sifter V8 to run.  The authors are encouraged to take the steps to digitally sign the macros in ASv8, which is one of the Macro Settings options for allowing VBA macros to operate, without allowing all macros to operate.

 2) Under the methods section, provide a table of the macros implemented in ASv8 with a description of what each macro does. Please provide the actual code for the VBA macros in a form that does not require “Enabling Macros” in Excel in order to review your code.  These could be text files added to the Zenodo item and named to identify the specific macro or it could just be supplemental material with this manuscript. 

 3) The version of ASv8 on Zenodo appears to be prepopulated with information from a query run for fipronil in the Main worksheet. Does “Query PubMed” macro erase this upon use?  This reviewer is assuming it does, but has not enabled Macros to find out, due to the scary message in Microsoft’s Trust Center warning not to enable Macros.

 4) Is the DSSTox database a (scientific)  “repository” as stated on page 13? A repository generally promotes preservation, versioning, provenance, and deposition.  A database or knowledgebase does not necessarily support these types of functions.

 Minor Issues:

 Across multiple figures, added text and boxes should be in a color that makes it clear they are not part of the screen capture. Often a bright red is used for this purpose.

 Page 4, line 1 under “Operation”: Remove “The” before “AS”.

 Page 4, line 1 under “Querying…”: add comma after “In this worksheet”.

 Page 4, paragraph 2, line 3 under “Querying…”: add comma after “For many literature projects”.

 Page 5, paragraph 2, line 3: add comma after “Using AS”.

 Page 6, last paragraph: define DTXSID at first use.  A quick google search brought up “Distributed Structure Searchable Toxicity Substance Identifier”.  This definition does not match the definition in the Abbreviations section; however, the google search may be wrong or old.

 Page 8, paragraph 2, line 5: a Mesh qualifier is usually in all caps and thus should be “THER USE”.

 Page 9, paragraph 2, line 2: add comma after “Word document”.

 Page 9, paragraph 3, line 1: grammatically incorrect sentence, but not sure how to fix it.

 Page 10, paragraph 2, line 2: add “the” before “user clicks”.

 Page 11, paragraph 2, line 4: add comma after “In this example”.

 Page 11, paragraph 3, line 4: add comma after “for the function”.

 Page 13, paragraph 2, line 3: replace “data set” with “dataset”.

 Figure 18: Suggest embellishing the figure legend to describe the steps and identify the order of the steps.

 Figure 19: Figure legend looks incomplete.  Not sure why it is starting “a and b. On the Abstract…”.  Feels like something was accidentally cut and/or pasted.

 Page 16, under “Understanding sets of chemicals”: Would suggest coming the first and second sentence for a better transition sentence.  Could start with “While the last section reviewed…, this section reviews approaches…”

 Page 17, paragraph 2, line 1: remove “The” before “AS” and add comma after “literature tool”.

 Page 17, paragraph 2, line 3: remove “the” before “AS”, which is on the next line.

 Page 17, paragraph 3, line 5: move the comma after “then” before “then”.

 Page 18, paragraph 1, line 5: remove “the” before “AS”.

 Page 19, paragraph 1, line 1: remove “The” before “AS”.

 Page 19, paragraph 3, line 1: add “a” before “large complex corpora”.

 Page 19, paragraph 4, line 1: remove “the” before “AS”.

 Page 19, paragraph 6, line 1: remove dashes and use commas, starting with putting a comma before “because”.

 Page 20, paragraph 3, line 1: remove “The” before “AS”.

Are the conclusions about the tool and its performance adequately supported by the findings presented in the article?

Partly

Is the rationale for developing the new software tool clearly explained?

Yes

Is the description of the software tool technically sound?

Partly

Are sufficient details of the code, methods and analysis (if applicable) provided to allow replication of the software development and its use by others?

Partly

Is sufficient information provided to allow interpretation of the expected output datasets and any results generated using the tool?

Yes

Reviewer Expertise:

scientific programming; bioinformatics, cheminformatics; systems biology

I confirm that I have read this submission and believe that I have an appropriate level of expertise to confirm that it is of an acceptable scientific standard, however I have significant reservations, as outlined above.

# Response

**Published:** 2026-05-20

**Authors:** Nancy Baker

**Affiliations:** Not Applicable, USA

Dear Reviewer,

 Thank you for the time and effort you put into reviewing the article and the tool. 

 Many things have changed since this manuscript was submitted.  The research arm of the EPA which funded Abstract Sifter has been dismantled and funding for work on the tool ceased.  I have continued on my own time to support the tool.  

 Here are my responses to the major issues:

 1. **Response: **Macros are a hurdle with developing Excel applications.  In response to this review, and user inquiries, I invested in getting a digital signing key.  There is one version of the tool on the download web site that is digitally signed.  However, that key only lasts for a year and I cannot sign another version until I renew it.  The cost is significant for me personally to support again.  I will add text to the manuscript and the user guide which lets the user know where to find the signed version and how to contribute if an organization would like then new versions signed. 

 Another security related issue is the use of Inkedit control. This control gives the Abstract Sifter the ability to colorize text in a form.  It's useful for screening lots of abstracts.  I have just found a way (thanks to clever contributors to the Internet help sites) to call the Inkedit control dynamically. This ability eliminates the irritating warning that used to come up. That version is now the downloadable version. 

**Comment: **2. The reviewer comment:

*2) Under the methods section, provide a table of the macros implemented in ASv8 with a description of what each macro does. Please provide the actual code for the VBA macros in a form that does not require “Enabling Macros” in Excel in order to review your code.  These could be text files added to the Zenodo item and named to identify the specific macro or it could just be supplemental material with this manuscript.*

**Response: **The code is now in Zenodo by module name.

 Note: The term “macros” does not really reflect the construction of an Excel macro-enable application.  The Abstract Sifter has code, VBA code, behind it. The file on Zenodo called vbacode.zip has all the code modules.  One can download that file and browse all the modules and the subroutines and functions in each module.  They are all in VBA code. The modules are named for their general functionality.

  Excel applications are challenging because they are worksheets, forms, and code all working together.  Abstract Sifter has thousands of lines of VBA code. It also has API calls that are often sent with a (secret) API key which has to be removed from the code before making it open.     

 I have extracted all the VBA code modules, forms, and sheets from Abstract Sifter into one zip file and added that to Zenodo. An interested user can read the code. If users wish to rebuild the application, they will need to get API keys under their own names.

**Comment: **3. Reviewer comments:

  The version of ASv8 on Zenodo appears to be prepopulated with information from a query run for fipronil in the Main worksheet. Does “Query PubMed” macro erase this upon use?  This reviewer is assuming it does, but has not enabled Macros to find out, due to the scary message in Microsoft’s Trust Center warning not to enable Macros.

***Author response: **Yes, the Abstract Sifter, like PubMed, is dynamic and running a query will erase the previous results unless the user decides to append results. It seems friendlier to have some results on the Main sheet, but I can stop this practice.*

**Comment: **4. Is the DSSTox database a (scientific) “repository” as stated on page 13? A repository generally promotes preservation, versioning, provenance, and deposition.  A database or knowledgebase does not necessarily support these types of functions.

***Author response:** I have replaced the term “repository” with “database” in reference to DSSTox and PubChem.*

 Minor Issues:

 Across multiple figures, added text and boxes should be in a color that makes it clear they are not part of the screen capture. Often a bright red is used for this purpose.

***Author response:** Thank you for this comment. Where possible, the added text has been changed from black to red color.*

**Comment: **Page 4, line 1 under “Operation”: Remove “The” before “AS”.

***Author response: **completed.*

**Comment: **Page 4, line 1 under “Querying…”: add comma after “In this worksheet”.

***Author response: **completed.*

**Comment: **Page 4, paragraph 2, line 3 under “Querying…”: add comma after “For many literature projects”.

***Author response: **completed.*

**Comment: **Page 5, paragraph 2, line 3: add comma after “Using AS”.

***Author response: **completed.*

**Comment: **Page 6, last paragraph: define DTXSID at first use.  A quick google search brought up “Distributed Structure Searchable Toxicity Substance Identifier”.  This definition does not match the definition in the Abbreviations section; however, the google search may be wrong or old.

***Author response: **completed.*

**Comment: **Page 8, paragraph 2, line 5: a Mesh qualifier is usually in all caps and thus should be “THER USE”.

***Author response: **completed.*

**Comment: **Page 9, paragraph 2, line 2: add comma after “Word document”.

***Author response:** completed.*

**Comment: **Page 9, paragraph 3, line 1: grammatically incorrect sentence, but not sure how to fix it.

***Author response: **The text has been simplified.*

**Comment: **Page 10, paragraph 2, line 2: add “the” before “user clicks”.

***Author response: **completed.*

**Comment: **Page 11, paragraph 2, line 4: add comma after “In this example”.

***Author response: **completed.*

**Comment: **Page 11, paragraph 3, line 4: add comma after “for the function”.

***Author response: **completed.*

**Comment: **Page 13, paragraph 2, line 3: replace “data set” with “dataset”.

*Author response: completed.*

 Figure 18: Suggest embellishing the figure legend to describe the steps and identify the order of the steps.

***Author response: **completed.*

**Comment: **Figure 19: Figure legend looks incomplete.  Not sure why it is starting “a and b. On the Abstract…”.  Feels like something was accidentally cut and/or pasted.

***Author response: ** thanks for this observation. I have added a. and b. to the figure for clarity.*

**Comment: **Page 16, under “Understanding sets of chemicals”: Would suggest coming the first and second sentence for a better transition sentence.  Could start with “While the last section reviewed…, this section reviews approaches…”

***Author response:** excellent suggestion. It now reads much better.*

**Comment: **Page 17, paragraph 2, line 1: remove “The” before “AS” and add comma after “literature tool”.

***Author response**: completed.*

**Comment: **Page 17, paragraph 2, line 3: remove “the” before “AS”, which is on the next line.

***Author response: **completed.*

**Comment: **Page 17, paragraph 3, line 5: move the comma after “then” before “then”.

***Author response: **completed.*

**Comment: **Page 18, paragraph 1, line 5: remove “the” before “AS”.

***Author response: **completed.*

**Comment: **Page 19, paragraph 1, line 1: remove “The” before “AS”.

***Author response: **completed.*

**Comment: **Page 19, paragraph 3, line 1: add “a” before “large complex corpora”.

***Author response: **completed.*

**Comment: **Page 19, paragraph 4, line 1: remove “the” before “AS”.

***Author response:** completed.*

**Comment: **Page 19, paragraph 6, line 1: remove dashes and use commas, starting with putting a comma before “because”.

***Author response:** completed.*

**Comment: **Page 20, paragraph 3, line 1: remove “The” before “AS”.

***Author response: **completed.*
